# Iron minerals within specific microfossil morphospecies of the 1.88 Ga Gunflint Formation

**DOI:** 10.1038/ncomms14890

**Published:** 2017-03-23

**Authors:** Kevin Lepot, Ahmed Addad, Andrew H. Knoll, Jian Wang, David Troadec, Armand Béché, Emmanuelle J. Javaux

**Affiliations:** 1 Laboratoire d'Océanologie et de Géosciences, Université de Lille, CNRS UMR8187, Cité Scientifique, SN5, 59655 Villeneuve d'Ascq, France; 2 Paléobiogéologie, Paléobotanique & Paléopalynologie, UR Geology, Département de Géologie, Université de Liège, 14 Allée du 6 Août B18, Quartier Agora, 4000 Liège, Belgium; 3 Unité Matériaux et Transformations, Université de Lille, CNRS UMR8207, Cité Scientifique C6, 59655 Villeneuve d'Ascq, France; 4 Department of Organismic and Evolutionary Biology, Harvard University, 26 Oxford Street, Cambridge, Massachusetts 02138, USA; 5 Canadian Light Source Inc., University of Saskatchewan, 44 Innovation Boulevard, Saskatoon, Saskatchewan, Canada S7N 2V3; 6 Institut d'Electronique, de Micro-électronique et de Nanotechnologie, CNRS UMR8520, Avenue Poincaré, 59655 Villeneuve d'Ascq, France; 7 Electron Microscopy for Material Science, University of Antwerp, Groenenborgerlaan 171, 2020 Antwerp, Belgium

## Abstract

Problematic microfossils dominate the palaeontological record between the Great Oxidation Event 2.4 billion years ago (Ga) and the last Palaeoproterozoic iron formations, deposited 500–600 million years later. These fossils are often associated with iron-rich sedimentary rocks, but their affinities, metabolism, and, hence, their contributions to Earth surface oxidation and Fe deposition remain unknown. Here we show that specific microfossil populations of the 1.88 Ga Gunflint Iron Formation contain Fe-silicate and Fe-carbonate nanocrystal concentrations in cell interiors. Fe minerals are absent in/on all organically preserved cell walls. These features are consistent with *in vivo* intracellular Fe biomineralization, with subsequent *in situ* recrystallization, but contrast with known patterns of post-mortem Fe mineralization. The Gunflint populations that display relatively large cells (thick-walled spheres, filament-forming rods) and intra-microfossil Fe minerals are consistent with oxygenic photosynthesizers but not with other Fe-mineralizing microorganisms studied so far. Fe biomineralization may have protected oxygenic photosynthesizers against Fe^2+^ toxicity during the Palaeoproterozoic.

## Abstract (web summary)

Fossil microorganisms older than 1.7 billion years are challenging to interpret due to their size, simple shapes, and alteration. Here, in 1.88 billion year old microfossils, the authors show a pattern of cellular preservation and internal iron nanominerals consistent with oxygenic photosynthetic bacteria.

Iron formations are widely distributed in Archaean (4–2.5 Ga) sedimentary successions^1^. After the Great Oxidation Event (GOE)^2^ at ca. 2.4 Ga, ferruginous conditions withdrew to deeper waters, although iron formation deposition and shallow-water ferruginous conditions recurred transiently at ∼1.9 Ga^3^. Unlike Fe^2+^, Fe^3+^ is essentially insoluble at pH >2, requiring iron transport as Fe^2+^. Fe isotopes of many iron formations support deposition through Fe^2+^ oxidation^1^, which could have proceeded via oxygenic photosynthesis^4^, chemotrophic metabolisms that use O_2_ (ref. 4) or its byproduct nitrate^5^ or anoxygenic photosynthesis^6^ that did not produce or require O_2_. Several redox-sensitive tracers^2^, including Mo isotopes^7^, have been interpreted as evidence for oxygenic photosynthesis as early as 2.9 Ga, and N isotopes support denitrification by 2.7 Ga^8^. These data collectively introduce a conundrum: if oxygenic photosynthetic bacteria (cyanobacteria) were present long before the GOE, why did not they rise to ecological prominence until the GOE and fully oxidize oceans and atmosphere until the Neoproterozoic Oxygenation Event (∼0.8 to 0.54 Ga)^2^? One possibility is suggested by comparative physiology; some of today's most common cyanobacteria show Fe intolerance that could have limited bacterial oxygenic photosynthesis in the Archaean^9^, and possibly later, during the Proterozoic (∼2.5 to 0.54 Ga)^10^.

During the interval from the GOE through the late Palaeoproterozoic resurgence of iron formations, a distinctive assemblage of microfossils—called Gunflint-type assemblages after their earliest discovered and most famous example^11^—dominate the fossil record. These assemblages occur most commonly either in or associated with iron-rich lithologies and commonly include shallow-water microbial mats that formed stromatolites^11^,^12^,^13^. In the 1.88 Ga Gunflint Iron Formation (Canada) and broadly coeval Frere Formation (Australia), stromatolites formed in ferruginous^11^,^13^,^14^ and sulfate-limited^15^ environments. The stratigraphic association of Gunflint-type microfossils and Palaeoproterozoic iron formation suggests that these fossils may record communities involved in iron metabolism or oxygen generation, but what role they played remains unknown.

Gunflint-type assemblages are dominated by filamentous microfossils less than ca. 2.5 μm across (*Gunflintia minuta*) variously interpreted as cyanobacteria (oxygenic photosynthetic bacteria)^11^,^16^,^17^ or chemotrophic bacteria that oxidized iron^11^,^14^,^18^ or sulfur^19^. The *G. minuta* morphospecies may include biologically distinct populations that, to date, cannot be distinguished because of post-mortem morphological convergence^18^,^20^. Abundant spheroidal microfossils assigned to the genus *Huroniospora* have also been interpreted either as cyanobacteria^11^ or as heterotrophs^21^. Infrared analyses of *G. minuta* and *Huroniospora* aggregates suggest that these microfossils preserved bacterial rather than archaeal or eukaryotic lipid derivatives^22^ but do not further constrain phylogeny or physiology. Carbon isotope analyses on individual *Huroniospora* and *G. minuta* are consistent with oxygenic photosynthesis by cyanobacteria^23^,^24^, but also with heterotrophy, anoxygenic photosynthesis or chemoautotrophy^25^. S isotopes of pyritized *Huroniospora* and *Gunflintia* indicate bacterial sulfate reduction by other microorganisms that consumed these cells^15^. Scarce filaments more than 3 μm across, including broad *Animikiea* and *Gunflintia grandis*, may include cyanobacteria, but, again, other interpretations are possible^11^,^17^. Colonial spheroids, some with intracellular inclusions, have also been assigned to cyanobacteria^16^,^26^. Scarce umbrella-shaped *Kakabekia* resemble extant microorganisms that may use ammonia as energy source^27^, while uncommon radiate structures (*Eoastrion*) resemble micro-colonies of the Mn- and Fe-oxidizing bacterium *Metallogenium*^18^. Thus, although Gunflint-type assemblages include a minor fraction of morphologically identified cyanobacteria^16^,^26^, conventional palaeontological and biogeochemical studies could not identify the dominant morphospecies unambiguously. Additional approaches are necessary to address phylogeny and metabolism.

Metabolic participation of Gunflint-type microorganisms in Fe deposition is suggested by microfossils mineralized by Fe^3+^ oxides^12^,^13^,^14^,^28^. Fe isotope and trace element compositions^14^, and Fe-rich depositional facies^11^,^14^ are all consistent with Fe oxidation by members of the Gunflint-type microbiota. However, microorganisms can mineralize iron passively (that is, irrespective of their metabolism), as cell surfaces complex and nucleate Fe minerals^4^. Co-culture experiments have shown that bacteria that become encrusted by Fe^3+^ minerals are not systematically those that oxidize Fe^2+^, which may have encrustation-preventing mechanisms^29^. Moreover, post-mortem Fe mineralization has been proposed for some Gunflint-type microfossil assemblages^12^. Thus, whether and how the Gunflint-type microfossils are related to Fe mineralization, and the metabolism they used, remain ambiguous^12^. A better understanding of the mineralogy and spatial relationships of iron deposited in association with Gunflint microfossils might, therefore, shed new light on their biological and diagenetic interpretation.

Here we study stromatolites from the lower ‘algal chert member' of the 1.88 Ga Gunflint Iron Formation, exposed at Schreiber Beach, Ontario, Canada. This locality displays the best preserved and least metamorphosed fossiliferous samples of Gunflint chert^11^,^12^,^30^. We document 32 microfossils (Supplementary Table 1) at the nanoscale (‘Methods' section) using a combination of scanning electron microscopy (SEM), focused ion beam (FIB) sectioning, scanning transmission electron microscopy (STEM) and scanning transmission X-ray microscopy (STXM). This distinguishes six morphological types among the dominant Gunflint microfossils, including two subtypes in *G. minuta* populations and two in *Huroniospora* populations. Three of the six differentiated morphospecies display dense internal iron mineralization. Hypotheses of post-mortem diagenetic origin must be taken seriously; however, our summary argument is that available data are best explained by iron mineralization-induced intracellularly within living cyanobacteria rather than via post-mortem processes.

## Results

### Stromatolite formation

Sample 70–85 is a chert characterized by cm-scale stromatolites formed by accretion and silicification of wavy organic laminae. The latter now essentially consist of dense microfossil populations (Supplementary Figs 1–3). Accretion occurred in a shallow, wave or current-influenced environment with input of ferrous iron (Supplementary Fig. 1 and refs 11, 14).

### Microfossil morphotypes

**Figure 1:**
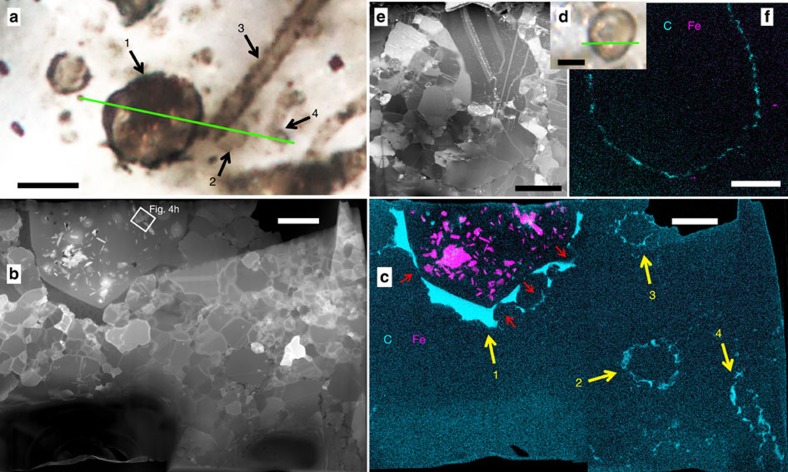
Iron in Gunflint microfossils. (**a**–**c**) 1: thick-walled *Huroniospora*, 2: thin-walled *Huroniospora*, 3–4: Type 1 (cell-free sheaths) *G. minuta*. (**d**–**f**) Thin-walled *Huroniospora*. (**a**,**d**) Multiplane photomicrographs. Scale bars, 5 μm. (**b**,**e**) STEM dark-field images of the FIB ultrathin sections cut along the green lines. Scale bars, 2 μm. (**c**,**f**) STEM maps of Fe (pink), C (cyan); corresponding maps of Si, O and S are shown in Supplementary Fig. 4. Scale bars, 2 μm. Fe minerals are highly concentrated inside thick-walled *Huroniospora* and nearly absent in or near Type 1 *G. minuta* and thin-walled *Huroniospora*. Red arrows in **c** indicate displacement of wall organic matter by quartz grains.

*Huroniospora* spheroids can be divided into at least two groups. The first group is characterized by thin walls (40–60 nm thick) and may be divided in two subgroups based on diameter (ca. 2.5 and 7–9 μm; Fig. 1, Supplementary Table 1 and Supplementary Figs 4–5). The second group comprises larger individuals (7–12 μm across) characterized by thicker walls (110–600 nm; Fig. 1a–c and Supplementary Fig. 6). In all *Huroniospora*, organic walls locally pinch and swell in correlation with the disposition of surrounding quartz crystals.

**Figure 2:**
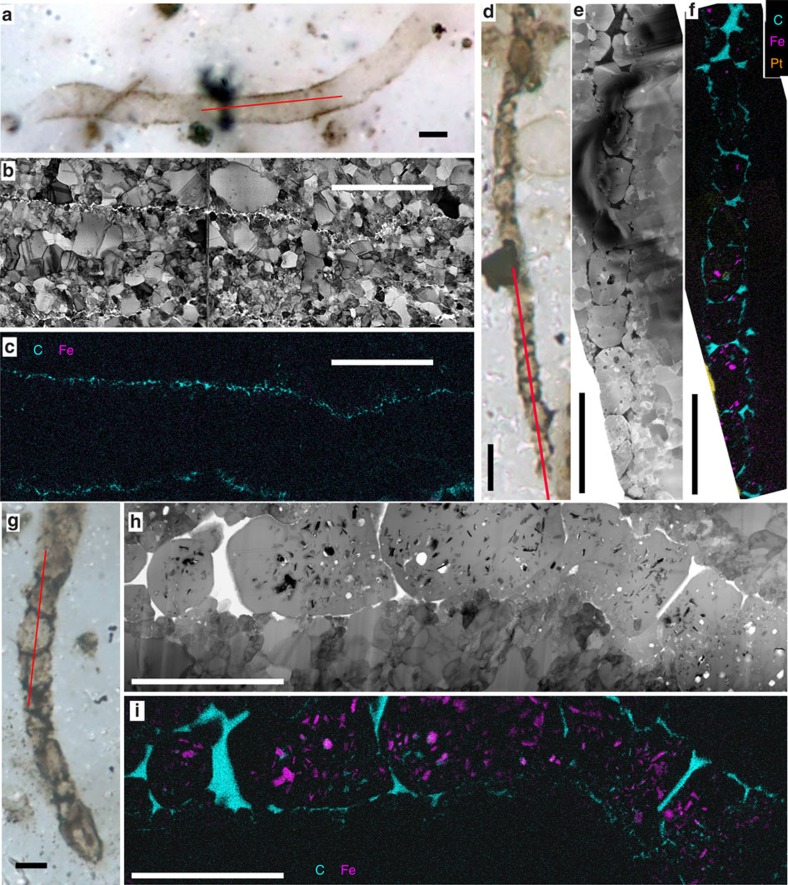
Iron in Gunflint filaments. Scale bars, 5 μm. (**a**–**c**) *Animikiea* of Type 1 (without cell remnants in sheath). (**d**–**f**) *G. minuta* of Type 2 (with cell-like segmentation). (**g**–**i**) Type 2 *G. grandis.* (**a**,**d**,**g**) Multiplane photomicrographs. (**b**,**e**,**h**) STEM bright- (**b**,**h**) and dark- (**e**) field images of the FIB ultrathin sections cut along the red lines. (**c**,**f**,**i**) STEM maps of Fe (pink), C (cyan) and preparation coatings (Pt: orange); corresponding maps of Si, O and S are shown in Supplementary Fig. 7. Fe minerals occur in *G. grandis* and *G. minuta* Type 2, but not in *Animikiea*. Fe minerals are absent in the vicinity of these microfossils.

Filamentous microfossils have been grouped into two main types based on structures observed in longitudinal section, with each divided into subtypes based on diameter. Type 1 filaments comprise tubular sheaths that do not contain cells or degraded cell material and with organic sheath thickness less than 150 nm (Figs 1a–c and 2a–c). Type 1 *G. minuta* comprises empty narrow (0.9–1.5 μm across) filamentous sheaths (Fig. 1a–c and Supplementary Figs 8–9), and Type 1 *Animikiea* comprises empty sheaths more than 3 μm across (Fig. 2a–c and Supplementary Fig. 10). The size of quartz crystals inside Type 1 filaments varies from several tens of nanometres to a few micrometres similar to crystals outside the filaments (Fig. 2b and Supplementary Fig. 9b). Type 2 comprises the filaments displaying internal organic contents, with transverse organic segments that complete organic coatings around cylindrical quartz grains (Fig. 2d–i), consistent with the original presence of chains of cylindrical (rod-shaped) cells. Type 2 *G. minuta* comprises the narrow (1.4–2.5 μm) segmented filaments (Fig. 2d–f and Supplementary Fig. 11) and Type 2 *G. grandis* comprises segmented filaments wider than 4 μm (Fig. 2g–i and Supplementary Fig. 12), as originally differentiated by Barghoorn and Tyler^11^. The abundance of organic matter in Type 2 filaments rules out origin by compression of the thin Type 1 sheaths. In particular, partly degraded Type 2 filaments (Supplementary Fig. 11c) form organic wisps too thick (∼1 μm) to correspond to collapsed Type 1 sheaths (Supplementary Figs 8–10). Most quartz crystals inside Type 2 filaments are as wide as the filaments and elongated along the filament's length. They are coarser than crystals outside the microfossils, and their size is uncorrelated with that of external, adjacent crystals. The distribution of segment lengths displays a sharp maximum at 3.5 μm in Type 2 *G. minuta* (Supplementary Fig. 13), and the average segment length is weakly anti-correlated with diameter in all Type 2 filaments.

*G. minuta* and *Huroniospora* comprise more than 90% of the studied microfossil assemblage, whereas *G. grandis* and *Animikiea* represent a few per cent at most. All microfossil morphotypes occur in close association with each other (Fig. 1a and Supplementary Fig. 3), although some regions are locally enriched in *Huroniospora* (thin- and thick-walled) while others contain mostly *Gunflintia* (for example, Supplementary Fig. 3d versus 3e).

### Fe mineralization of microfossils

**Figure 3:**
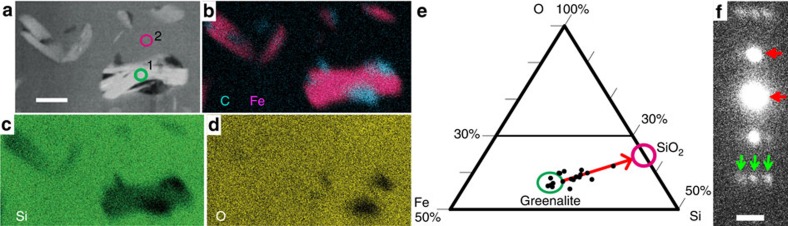
Intra-microfossil greenalite. (**a**–**d**) STEM of greenalite in quartz. (**a**) Dark-field image. Scale bar, 200 nm. Circles numbered 1 and 2 outline target areas of EDXS and EELS spectra (displayed in Supplementary Fig. 15 with corresponding numbers). (**b**–**d**) EDXS mappings of Fe (pink in **b**), organic C (cyan in **b**), Si (green in **c**), O (yellow in **d**) in the region in **a**. The crystals in the green circle traverse the FIB section, allowing EDXS analyses without quartz interference, as confirmed by Si EELS spectra. (**e**) Ternary Fe–Si–O (atomic %) plot derived from EDXS spectra (Supplementary Fig. 15c): quartz-free greenalites (including that numbered 1 in **a**) plot in the green circle, whereas other crystals plot on a mixing line with quartz (pink circle), indicating that all greenalite crystals have a similar composition. The Si/O ratio of 0.32 (*n*=4 quartz-free crystals) is consistent with the general formula of greenalite: Si_2_O_5_(OH)_4_(Fe^2+^,Fe^3+^)_2–3_. EELS spectra display a Fe^3+^/Fe_total_ <4.5‰ (Supplementary Fig. 15e), indicating a composition close to Si_2_O_5_(OH)_4_(Fe^2+^)_3_. (**f**) SAED pattern recorded on the region numbered 1 in **a**. Scale bar, 1 nm^−1^. Arrows indicate lattice spacing diagnostic of greenalite: 7.2 Å (0,0,1) planes (red), and 23 Å superlattice (green).

**Figure 4:**
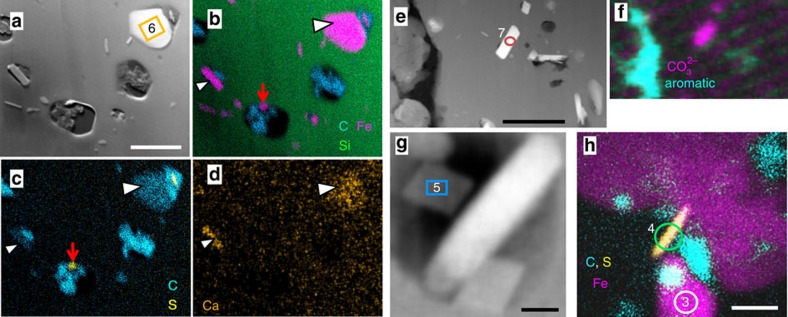
Intra-microfossil siderite and Fe^2+^ sulfides. (**a**) STEM dark-field image. (**b**–**d**) EDXS mappings of **a** showing quartz (Si: green in **b**), siderite (Fe: pink in **b**, minor Ca: orange in **d**, arrowheads), and Fe+S minerals (S in yellow in **c**, red arrows in **b**,**c**) that are systematically nanoscale and embedded in organic matter (C: cyan in **b**,**c**). Scale bar, 500 nm. (**e**,**f**) STEM dark-field image (**e**) and STXM mapping (**f**) of **e** showing carbonate (pink) and aromatic carbon (cyan). The rod-shaped (red circle) crystal is siderite. Scale bar, 500 nm. (**g**) STEM dark-field image showing rhombohedra of siderite (for example, blue box) associated with greenalite (white rod). Scale bar, 50 nm. (**h**) EDXS mapping of a nanoscale Fe^2+^ and S (yellow) crystal embedded in organic carbon (cyan) between two greenalite (Fe: pink) crystals. Scale bar, 100 nm. Numbered boxes and circles in **a**,**e**,**g**,**h** outline target areas of EDXS spectra displayed in Supplementary Figs 15 and 16 with corresponding numbers.

Thick-walled *Huroniospora* (Fig. 1a–c and Supplementary Figs 6 and 14a–c), *G. minuta* Type 2 (Fig. 2d–f and Supplementary Fig. 11) and *G. grandis* Type 2 (Fig. 2g–i and Supplementary Figs 12 and 14d–f) display dense internal Fe mineralization. Intra-microfossil Fe nanocrystals occur mainly as plate-like greenalite (Fig. 3, measured approximate composition Si_2_O_5_(OH)_4_Fe^2+^_3_, Supplementary Fig. 15), together with rhombohedron- or rod-shaped siderite (Fe^2+^CO_3_, Fig. 4), and rare Fe^2+^ sulfides systematically embedded in organic matter (Fig. 4 and Supplementary Fig. 16). Image analysis of thick-walled *Huroniospora* (‘Methods' section) yielded an estimated range of 10^8^–10^9^ Fe atoms per μm^3^ of cell, whereas one *G. grandis* filament displayed 8.6 × 10^8^ Fe atoms per μm^3^. ‘Background' Fe minerals in the quartz matrix less than ∼10 μm away from microfossils occur as plate-like (that is, likely greenalite) nanocrystals smaller than intra-microfossil crystals. The ‘background' Fe-nanocrystal concentration amounts to less than 1.5 × 10^6^ Fe atoms per μm^3^. In contrast, thin-walled *Huroniospora* (Fig. 1 and Supplementary Fig. 5), *G. minuta* Type 1 (Fig. 1a–c and Supplementary Figs 8–9) and *Animikiea* (Fig. 2a–c and Supplementary Fig. 10) displayed at most a few plate-like (likely also greenalite) intra-microfossil Fe nanocrystals. These were less abundant than the Fe nanocrystals in the surrounding ‘background' (Supplementary Figs 5b and 10e).

### Stromatolite composition and mineralogy

Deciphering the origin and fate of organic matter and Fe minerals in microfossils requires complementary characterization of these compounds in the surrounding matrix. In the matrix surrounding all FIB-sectioned microfossils, STEM and STXM could not detect ‘background' organic matter (for example, Figs 1 and 2). Neither was siderite observed outside microfossils. Pyrite (FeS_2_) occurs in scarce patches where it systematically replaces organic structures of microfossils and overgrows such pyritized microfossils, and as partial replacement of sediment grains (Supplementary Fig. 17a–e). Ankerite [(Ca,Mg,Fe^2+^, Mn)CO_3_] rhombohedra a few hundred micrometres in maximum dimension occur mostly in veins, while a small fraction is heterogeneously distributed in the chert matrix (Supplementary Fig. 17d–f). All ankerites systematically display chemical zonation with a central Ca-, Mg- and Mn-rich core and Fe-rich overgrowth. Some ankerite embedded in quartz replaced parts of pyritized sediment grains, and includes pyrite. This shows that ankerite grew after and replaced other minerals within the stromatolites, consistent with observations that ankerite is a later diagenetic phase likely replacing earlier siderite, greenalite and chert in various Gunflint facies^11^,^31^. Finally, Fe-oxide minerals, such as haematite (Fe^3+^_2_O_3_) or magnetite (Fe^2+^Fe^3+^_2_O_4_), were not observed. Poorly crystalline Fe^3+^-(oxy)hydroxides occurred only in open fractures (Supplementary Fig. 17g) and are accordingly considered as post-exhumation weathering products.

## Discussion

Nanoscale observations of the organic structures and of their quartz matrix provide new constraints on the origin of the dominant Gunflint microfossils. First, in *Huroniospora*, wall thickness discontinuities have been interpreted as initial features of the cells such as wall reticulation, budding or opening of cells and used to argue against cyanobacterial origin^21^. We show that these discontinuities follow the local morphology of embedding quartz crystals, supporting the alternative view that these textures formed through post-mortem displacement of organic matter^16^. Diameter heterogeneities are consistent with previous hypotheses that *Huroniospora* represents a composite morphospecies^11^,^21^, and wall thickness measurements support the recognition of at least two distinct *Huroniospora* populations. Cell wall thickness, as well as delicate wall internal structures, can be diagnostically preserved in the rock record^32^. Nevertheless, it has been hypothesized that thick-walled *Huroniospora* formed through post-mortem accretion of organic matter onto thin-walled *Huroniospora* on the basis that the former were observed in kerogen-rich regions, whereas the latter were observed in kerogen-poor regions^21^. Actually, however, both occur together in microfossil-rich regions where ‘background' kerogen is absent (Fig. 1a–c and Supplementary Figs 3d and 6). Moreover, preservation of microfossils in 3D required silica encapsulation and impregnation^33^, which may not have left space for the required threefold to tenfold increase in wall thickness. The absence of overlap in wall thickness ranges argues that the thicker-walled (110–600 nm) *Huroniospora* could not form through thickening of thinner-walled (40–60 nm) *Huroniospora*. Hence, this difference reflects distinct precursor wall structures.

Similarly, STEM reveals distinct morphospecies among *Gunflintia* filaments. Type 2 *G. minuta* and *G. grandis* display cell-like structures, some of which have been interpreted as cyanobacterial in origin^16^,^26^. In contrast, Type 1 *G. minuta* only show tubular sheaths^11^,^18^. Previous studies could not distinguish cell-like structures from degraded sheaths^20^. Here we show that the abundance of organic matter in Type 1 sheaths is too low to produce Type 2 cell-like structures through displacement of organic matter. Hence, Type 2 represents either a variant of Type 1 that preserved cellular material or a distinct species. The sharp maximum in segment length distribution in Type 2 *G. minuta* argues for the preservation of ca. 3.5 μm long, 1.4–2.5 μm wide rod-shaped cells in septate filaments. Similarly, *G. grandis* could have preserved ca. 5 μm long and ca. 4.5 μm wide cells (although length is less constrained than for *G. minuta* due to the small number of measurements). Interestingly, the lower curvature of *G. minuta* filaments compared to broader Gunflint filaments has been used to suggest that the former had cells with higher length/diameter that provided them with higher flexural rigidity in spite of their small diameter^17^. This is consistent with our observation of elongated segments in *G. minuta* and the lower-segment elongation of broader filaments (Supplementary Fig. 13). The elongation of Type 2 *Gunflintia* segments is consistent with cell shapes of cyanobacteria^17^,^34^, anoxygenic phototrophic bacteria^35^, methanotrophic bacteria^36^, chemo-organoheterotrophic (using carbon sources as donors and acceptors of electrons) bacteria^37^ and sulfate-reducing bacteria^38^, as well as chemotrophic sulfur- and iron-oxidizing bacteria^19^,^39^. Cell-like segment length heterogeneity can be explained by organic matter displacement after the death of all cells in the filaments, by locally unpreserved septa, by fossilization of smaller necridial (decaying) cells together with larger vegetative cells^40^, by heterogeneity in vegetative cell length (for example, in cyanobacteria^34^), or by cyanobacterial specialized cells^16^.

In addition, the cell diameters in most Type 2 *G. minuta*, and in all *G. grandis* filaments exceed the maximum of 1.5 μm observed in filamentous anoxygenic phototrophic bacteria^35^ and in filamentous chemo-lithoautotrophic bacteria that derive energy from Fe oxidation (*Leptothrix*)^37^,^39^. Although post-mortem filament shrinkage could have occurred^17^, diameter increase should be associated with systematic tearing of the sheaths or cell walls, which was not observed here. The diameters and elongated cellular structures of Type 2 *G. minuta* and *G. grandis* are consistent with cyanobacteria^17^, chemotrophic S-oxidizing^19^ and S-reducing^38^ bacteria and methanotrophic bacteria depositing iron extracellularly^36^. *G. minuta* (unlike *G. grandis*) is also small enough to represent chemo-organoheterotrophic bacteria like *Sphaerotilus*^37^,^39^. In contrast, most filamentous eukaryotes are larger^41^ and display a distinct lipid profile^22^. Type 1 *G. minuta* is narrower (0.9–1.5 μm) and could also represent Fe-oxidizing chemolithoautotrophs (*Leptothrix*-like) or anoxygenic photosynthesizers in addition to cyanobacteria and aforementioned chemotrophs.

Gunflint microfossils are intimately associated with Fe minerals that may provide constraints on their nature and metabolism. This requires deciphering the multistage iron deposition and transformation sequences that occurred in Gunflint rocks (Supplementary Fig. 18 and Supplementary Discussion). In shallow-water fossiliferous stromatolites and in deeper-water iron formations of the Gunflint Iron Formation, Fe-isotope ratios^14^ support the hypothesis that most of bulk-rock iron precipitated through partial oxidation of ferruginous seawater (refs 1, 14 and Supplementary Discussion). Measured Fe-isotope values are consistent with Fe oxidation by chemotrophic bacteria^14^ or cyanobacteria^42^ as well as abiotic oxidation^1^. Subsequent Fe reduction coupled to organic matter oxidation driven by bacteria^43^ and/or thermal energy^44^ could have formed the greenalite+siderite+Fe-sulfide+ankerite assemblage observed in our stromatolite sample similar to the assemblage observed in some Gunflint iron formations^1^,^14^. Many Gunflint-type microfossil assemblages have been affected by oxidizing groundwater during burial leading, to complete, non-specific replacement of Fe minerals and organic matter by haematite^11^,^12^,^13^. The sampled locality does not display petrographic evidence of hydrothermal alteration and displays the best molecular preservation of the Gunflint Iron Formation^30^. Indeed, in this relatively pristine sample, microfossils are either organically preserved or pyritized; none have been replaced by haematite (Figs 1 and 2 and Supplementary Figs 2–6). Intra-microfossil Fe^2+^ minerals, granular pyrite, ankerite and pyritized microfossils have not been oxidized except in recently opened cracks (Supplementary Fig. 17). No Fe-oxide minerals were detected in the studied sample except in recently weathered cracks. Moreover, oxidation of pyritized microfossils cannot be invoked here to explain the occurrence of organic microfossils with internal Fe minerals. Indeed, pyritization nearly obliterates organic structures^15^, which is inconsistent with observed preservation. Taken together, these observations support the view that the observed greenalite+siderite+Fe-sulfide assemblage has not been altered by oxidizing fluids and could have formed through reduction of primary, Fe^3+^ minerals.

The observed greenalite, siderite and sulfides could have formed through *in situ* reduction of intra-microfossil Fe^3+^ minerals, through intra-microfossil precipitation of Fe^2+^ minerals, or through dissolution–reprecipitation of extra-microfossil Fe minerals. Thick-walled *Huroniospora* and *G. grandis* host 10^8^–10^9^ Fe atoms per μm^3^, which is 2–4 orders of magnitude higher than in non-mineralizing bacteria (*E. coli*: ∼10^5^ and *Synechocystis* cyanobacteria: ∼10^6^ Fe atoms per μm^3^)^45^. Hence, these microfossils either concentrated iron *in vivo* through intracellular biomineralization, or in their decaying cytoplasm. In recent microorganisms, *in vivo* internal Fe biomineralization can be demonstrated when cytoplasmic ultrastructures are preserved^40^, when minerals occur within intracytoplasmic vesicles^46^,^47^,^48^ or when there is a persistence or increase of metabolism despite systematic Fe mineralization^47^,^49^. Fe mineralization may alternatively be explained as post mortem in recent DNA-free cells^5^ that show extensive mineral replacement of cytoplasmic ultrastructures together with cell wall mineralization^50^,^51^. Such diagnoses are difficult in Gunflint microfossils as cytoplasmic ultrastructures are not preserved and initial Fe precipitates were likely transformed. However, the general distributions of Fe minerals in the stromatolite help discuss *in vivo* and post-mortem hypotheses for Fe mineralization.

Post-mortem Fe-mineralization patterns are governed by the structure of decaying microfossils, the external physical environment and the metabolism and structure of heterotrophic microorganisms. These factors are discussed below.

First, the macromolecular components of cell walls bind metallic ions such as iron in living as well as in long dead cytoplasm-free cells^52^,^53^. Moreover, the wall of a dead cell can be modelled as a double-diffusion boundary where externally sourced Fe-rich fluids meet the organic-rich microenvironment imposed by decaying cytoplasm: this boundary favours Fe precipitation (Supplementary Fig. 19). Thus, aqueous Fe migration onto decaying microorganisms generally mineralizes walls preferentially. Indeed, recent intra-microfossil, post-mortem Fe-silicate^50^,^54^ and Fe-oxide^5^,^51^,^54^ precipitations are systematically associated with abundant extracellular and cell wall precipitates. Alternatively, Fe may accrete onto/into decaying microorganisms through transport in colloidal rather than aqueous form. However, solid organic matter has a strong affinity for Fe^3+^ colloids^55^. Hence, similar to aqueous Fe^2+^, these colloids should strongly bind to cell walls through which they diffuse before they can reach the decaying cytoplasm. In contrast, post-mortem Fe silicates observed in ancient microfossils preferentially encrust the inner and/or outer wall surfaces^56^. Post-mortem Fe-bearing carbonates (ankerite) and oxides (magnetite) also preferentially encrust microfossil walls and are abundant in the surrounding matrix^57^. After post-mortem Fe mineralization of wood, similar Fe minerals are observed inside cells, in/on their wall and outside cells in the same sample (for example, Fig. 12 in ref. 58). Post-mortem Fe mineralization of bone cells also occurs preferentially in/on walls^59^. Furthermore, pyritization of Gunflint microfossils similarly resulted of post-mortem Fe mineralization of microfossil walls and of the matrix surrounding microfossils (ref. 15 and Supplementary Figs 17a–c and 20). In contrast, greenalite+siderite in the observed Gunflint microfossils are only scattered at the centre of cells and do not concentrate on/in/near the cell wall or outside the microfossils (Figs 1 and 2).

Second, preservation of specific cellular structures (walls, membranes and/or cytoplasm) may have allowed Fe mineralization. Cell-preserving Type 2 *Gunflintia* is Fe mineralized, whereas cell-free sheaths (Type 1 *Gunflintia* and *Animikiea*) remained iron free. This is, however, at odds with the observed affinity of cell-free sheaths for iron^60^. In addition, the 3D preservation of adjacent *Huroniospora* types (Fig. 1) shows that they were similarly encapsulated in SiO_2_ alive or soon after death with cytoplasmic content preventing flattening and folding (Fig. 1a–f). However, Fe mineralization only affected the thick-walled *Huroniospora*, suggesting that cell wall preservation alone could not have mediated post-mortem Fe mineralization. Interestingly, Fe-mineralized morphospecies include intra-microfossil organic matter (Figs 1 and 2 and Supplementary Fig. 6). Although a large fraction of this organic matter may result from the displacement of cell wall fragments (Supplementary Fig. 6), it may also contain remnants of membrane and/or cytoplasmic molecules as suggested by carbon isotopes in Neoproterozoic cyanobacteria^24^. These molecules may have permitted post-mortem Fe mineralization. The distribution and abundance of intra-microfossil organic matter show no clear relationships with those of Fe minerals in support of this scenario. Moreover, this scenario requires again that chemical compounds allowed post-mortem Fe mineralization of internal parts/contents of microfossils but not of wall organic matter, which, as discussed above, has not been observed *in vitro*, or in other microfossils mineralized with Fe silicates, Fe oxides or Fe carbonates, or in pyritized Gunflint microfossils. Indeed, after cell death intracellular molecules usually leak in the surrounding medium (which cannot be prevented where silica is permeable enough to allow Fe counter diffusion) and/or polymerize (for example, ref. 61) or adsorb (for example, ref. 62) on cell walls. The low abundance of greenalite and siderite outside microfossils, and their absence in/on the walls of heavily Fe-mineralized microfossils imply that cytoplasmic organic molecules redistributed very little into/outside cell walls, and/or that they had little effect on Fe mineralization.

Third, post-mortem biodegradations can change the effect of organic molecules on iron and favour Fe remobilization at the same time. Although cytoplasmic contents (amino acids, proteins and nucleic acids) are the most labile food source for Fe-mineralizing heterotrophs such as sulfate- or iron-reducers, their consumption requires breaching of cell walls by the heterotrophs. Breaching of cells leaks cytoplasmic contents and feeds extra-microfossil heterotrophs. In this case, unless the Fe source was only intracellular (that is, biominerals), Fe-mineral byproducts of heterotrophy will precipitate inside as much as outside microfossils (Supplementary Figs 21 and 22). In some rocks, intra-microfossil pyrites occur without systematic wall pyritization, but this is always associated with abundant extra-microfossil pyrite (for example, ref. 63). Intra-microfossil pyrites are most commonly found in pyritic clayey matrices where microfossils have been flattened and torn by sediments, consistent with leakage of cytoplasmic content^64^. Intra-microfossil pyrites sometimes occur in water-conducing wood cells that have been open to fluids and heterotrophs, and are again associated with wall Fe mineralization^64^,^65^. However, greenalite displays a two-order of magnitude enrichment in specific microfossil morphospecies compared to the ‘background' matrix, and siderite was only observed in greenalite-rich microfossils. Thus, post-mortem Fe mineralization of microfossils by heterotrophs (for example, Supplementary Figs 21 and 22) is not consistent with the scarcity of greenalite and the absence of siderite outside microfossils, and the absence of Fe minerals on their walls. Interestingly, post-mortem pyritization did occur within the formation (it affected all microfossil morphospecies and extensively replaced organic matter), but was confined to specific patches in Gunflint stromatolites (Supplementary Figs 17a–c and 20 and ref. 15). Microfossils with preserved organic walls, including those with greenalite+siderite, may have been sheltered from heterotrophic bacterial sulfate reduction, possibly due to earlier and/or more impermeable silicification. Fe^2+^ diffusion and/or other heterotrophic metabolisms, such as iron reduction, may similarly have been disabled by early silicification, preventing continuing post-mortem Fe mineralization. Accordingly, the preservation of protein-derived amide-bearing molecules in Gunflint microfossils at the studied (Schreiber) locality support the hypothesis that early silicification favoured the preservation of organic matter against heterotrophic consumption^30^.

Fourth, known post-mortem Fe mineralizations do not affect specific types/morphospecies of microfossils or all microfossils of a given type. In recent cyanobacterial mats^51^ and in chemotrophic cultures^5^, heavily Fe-mineralized dead cells are intimately associated with their viable counterparts. Similarly, Fe mineralization of water-conducing wood cells is not systematic in a given sample (Fig. 12 in ref. 58). Pyritizations of Gunflint^15^ and other^64^ microfossils are not systematic or morphospecies-specific. In contrast, thick-walled *Huroniospora* and *G. grandis* are systematically mineralized with abundant greenalite+siderite, whereas, for example, thin-walled *Huroniospora* are extremely depleted in Fe minerals.

Finally, a heterogeneous preservation of intra-microfossil Fe minerals in specific morphospecies could not be controlled by the grain size of SiO_2_. Indeed, the quartz crystal size is similar within unmineralized Type 1 and Fe-mineralized Type 2 *G. minuta* (Supplementary Fig. 9b versus 2e). Micrometre-scale quartz coarser than the surrounding matrix occurs in both thick- and thin-walled *Huroniospora* (Fig. 1b versus 1e).

*In vivo*, microorganisms sometimes favour intracellular (in cytoplasm) over epicellular (on cell wall) or extracellular Fe biomineralization^40^,^47^,^49^,^66^,^67^. Although extra- and epicellular Fe-silicate biomineralization is known^68^, intracellular (that is, *in vivo*) greenalite precipitation is not. Similarly, intracellular siderite is not known. Instead of precipitating directly, intra-microfossil greenalite+siderite+Fe-sulfides could have derived from *in situ* transformation (reductive recrystallization) of intracellular Fe^3+^ or mixed Fe^2+^–Fe^3+^ biominerals (Supplementary Fig. 23). The scarce extra-microfossil greenalite could similarly have formed after minor extracellular biominerals. Recrystallization through Fe^3+^ reduction is consistent with the Fe isotope record of Gunflint stromatolites^14^ (Supplementary Discussion). Initial biominerals could have been Fe^3+^-oxyhydroxides or -phosphates (for example, cyanobacteria^40^,^49^, euglena algae^47^), Fe^2+^–Fe^3+^-oxides or -sulfides (for example, magnetotactic bacteria^46^) or Fe^2+^–Fe^3+^-phosphates (Fe-reducing bacteria^67^).

Altogether, the Fe-mineralization pattern of studied Gunflint microfossils is difficult to reconcile with post-mortem processes inferred from properties of cellular materials and from other fossil occurrences. Indeed, the observed Fe mineralization requires the existence of conditions (that is, most likely, organic molecules) that favour Fe precipitation inside, but not outside or in/at the wall of microfossils. The existence of such conditions remains to be tested with *in vitro* fossilization/diagenesis experiments. *In situ* thermal reduction of intracellular Fe biominerals provides a consistent, though indirect route for the formation of the observed Fe-mineral assemblage and its distribution. The following paragraphs discuss possible palaeontological implications of this interpretation.

In Gunflint-type assemblages, the empty Type 1 *G. minuta* sheaths have been interpreted as microaerophilic chemotrophic Fe-oxidizing bacteria based on morphological similarity with modern *Leptothrix*, which forms Fe-encrusted sheaths where cells have left or have been lysed^4^,^14^,^18^. Haematite associated with those filaments has traditionally been used to support this interpretation^18^. This haematite has, however, been proposed to represent a post-mortem feature of all microfossil types in Gunflint samples altered by oxidizing groundwater^12^. This is consistent with the absence of Fe minerals in/on Type 1 *Gunflintia* in our relatively pristine sample. In the absence of Fe mineralization and diagnostic morphological constraints, this morphospecies could represent various other microorganisms, including heterotrophs^37^ or phototrophs^11^. Nevertheless, some Gunflint microfossils may preserve organic carbon and primary haematite^14^ and should be investigated at the nanoscale for evidence of biomineralization.

In combination with morphology, the intra-microfossil Fe minerals help interpret the nature of thick-walled *Huroniospora* and Type 2 *Gunflintia*. Microaerophilic chemolithotrophic Fe-oxidizing bacteria and anoxygenic photoautotrophic Fe-oxidizing bacteria precipitate iron epi- but not intra-cellularly^4^,^6^. Hence, the intra-microfossil Fe mineralization is not consistent with known microaerophilic and photoautotrophic Fe-oxidizing bacteria. Nitrate-reducing anaerobic bacteria inducing Fe^2+^ oxidation crystallize Fe^3+^ minerals on their outer surface, inside their periplasm and in their cytoplasm^5^. The intracellular mineralization occurs after complete external encrustation in the nitrate-reducing bacteria^5^, contrasting with the absence of Fe minerals near the cell walls of the microfossils. The only microorganisms known to produce intracellular Fe minerals and possible counterparts for the observed microfossils are: magnetotactic bacteria^46^, dissimilatory iron-reducing bacteria^67^, Fe-polyphosphate-accumulating bacteria^69^ and oxygenic phototrophs including cyanobacteria^40^,^49^ and eukaryotic algae^47^,^66^. Akaganeite (Fe^3+^OOH) crystals formed intracellularly by living cyanobacteria have sizes and spatial distributions comparable to those of greenalite and siderite in Gunflint fossils, and may accordingly have been their precursors^49^.

Fe-mineralized thick-walled *Huroniospora* are too large (7–12 μm across) to be attributed to known intracellularly Fe-mineralizing microorganisms other than cyanobacteria and algae. Known anoxygenic photosynthetic Fe-oxidizing bacteria, nitrate-reducing bacteria, microaerophilic Fe-oxidizing bacteria, Fe-reducing bacteria, magnetotactic bacteria and Fe-phosphate-accumulating bacteria all have cells smaller than two micrometres^4^,^5^,^6^,^29^,^46^,^67^,^69^. In addition, the thick wall of these *Huroniospora* is consistent with cyanobacteria^70^ and algae, but not the unicellular Fe-oxidizing, Fe-reducing, magnetotactic and phosphate-accumulating bacteria, which are all thin walled. Similarly, methanotrophic, chemo-organoheterotrophic bacteria or chemotrophic S-oxidizing (or S-reducing) bacteria do not mineralize iron intracellularly and hence are not consistent with Type 2 *Gunflintia*. Taken together, the distribution of Fe minerals and morphology of thick-walled *Huroniospora* and Type 2 *Gunflintia* are best explained by cyanobacterial or algal phototrophs. As noted earlier, size and organic geochemistry favour interpretation of these populations as bacterial, favouring assignment of *G. minuta* to cyanobacteria. This actualistic assignment relies on the fact that intracellular Fe minerals have not, to our knowledge, been reported in other, morphologically similar microorganisms. This is strengthened by the fact that large, thick-walled spheres like *Huroniospora* are also unknown among the well-studied non-oxygenic microorganisms that mineralize iron extracellularly through iron oxidation or reduction.

The cyanobacterial and/or algal assignment of Fe-mineralized Gunflint microfossils imply that they possessed photosynthetic ability. Instead of oxygenic photosynthesis, cyanobacteria may have performed anoxygenic photosynthesis using H_2_S (ref. 70) (so far, photoferrotrophic metabolism^6^ has never been observed in cyanobacteria). Before it could be used for anoxygenic photosynthesis, H_2_S would have precipitated all iron into sulfide minerals, which is inconsistent with observed non-sulfide Fe minerals. Moreover, cyanobacteria can metabolize heterotrophically during the night but favour photosynthesis when light is available^70^. In their shallow-water environment, the Gunflint microfossils we interpret as cyanobacteria could have performed oxygenic photosynthesis during the day. In these conditions, Fe oxidation by O_2_ likely triggered iron mineralization, although the processes leading to intracellular biomineralization remain obscure^40^,^49^.

Oxygenic phototrophs have a high iron demand and are able to maintain higher intracellular Fe concentrations than non-photosynthetic bacteria^45^. Fe^2+^ is, however, particularly toxic to oxygenic photosynthesizers, as it reacts with O_2_ to form radicals, generating intracellular oxidative stress^9^,^40^,^45^. Elevated dissolved Fe^2+^ could thus have limited oxygenic photosynthesis before the GOE 2.4–2.3 Gyrs ago^9^. Similarly, the renewed incursion of Fe^2+^ into the photic zone, documented by 1.9–1.8 Ga iron formations such as the Gunflint^3^, could once again have inhibited oxygenic photosynthesis in Fe-rich environments. Some cyanobacteria and photosynthetic eukaryotes that tolerate, and sometimes also depend on high iron concentrations, mineralize Fe intracellularly^40^,^47^,^49^,^66^. The Fe mineralization of thick-walled *Huroniospora* and Type 2 *Gunflintia* support that, 1.88 Ga ago, oxygenic photosynthesizers had to cope with elevated iron concentrations even in shallow-water environments. These microorganisms, abundant in the stromatolites, would already have evolved mechanisms to alleviate Fe^2+^-induced intracellular oxidative stress through biomineralization.

The observed internal Fe minerals link specific 1.88 Ga fossil microorganisms to Fe mineralization. Moreover, this link supports intracellular biomineralization. Intracellular biomineralizations of iron^40^,^46^,^47^,^49^,^66^,^67^,^69^ and other elements have been documented in extant bacteria. These represent powerful targets to decipher microbial evolution by supplementing morphological features of microfossils with mineral signatures, but remain elusive in the fossil record due to their sub-micrometre size. The inferred intracellular biomineralization combined with novel nanoscale distinction of original cellular structures in Gunflint microfossils support that some of the most abundant microfossils of the Palaeoproterozoic were Fe-tolerant oxygenic photosynthesizers rather than lithotrophic, heterotrophic or anoxygenic photosynthetic bacteria.

## Methods

### Optical microscopy and multiplane images

Photomicrographs of petrographic thin sections were shot using an Olympus BX50 microscope using a × 40 objective (Supplementary Figs 3h and 6a) and an Olympus BX60 microscope using a × 100 objective (NA: 0.9) (all other figures). Photomicrographs from different focal planes (for example, Supplementary Fig. 2) were combined into ‘multiplane images' using a weighted average algorithm (CombineZP software by Alan Hadley). Focal depth was measured using a X-Y-Z automated stage with <1 μm-depth resolution. Microfossils embedded in the quartz matrix, between the thin section surface and 10 μm below, were selected for FIB sectioning.

### FIB

FIB ultrathin sections ∼20 × 10 μm large were prepared to analyse microfossils using transmission electron microscopy (TEM), STEM and STXM. We prepared ca. 120 nm-thin sections for STEM, TEM and STXM, and ∼70 nm sections for electron energy loss spectroscopy (EELS) and energy dispersive X-ray spectrometry (EDXS) analyses of nanocrystals. Petrographic thin sections were coated with ca. 50–100 nm of gold palladium. Microfossils were precisely localized using a FEI XL30 field-emission SEM (at Cat-μ Liège) operated at 10 mm and 10 kV. SEM analyses of Fe-bearing phases were performed (at Université de Lille) using a FEI Quanta 200 SEM equipped with a Bruker Quantax EDXS, operated at 10 mm and 30 kV. FIB sections were extracted from the petrographic sections using a FEI strata Dual Beam 235 FIB (IEMN Lille) and a HELIOS 600 nanolab DUAL Beam (CP2M Marseille). The top surface of each region of interest was protected with a strip of platinum ∼25 × 2 × 2 μm in dimensions. Material on each side of the region of interest was removed by a gallium ion beam operating at 30 kV, 7 nA. Then, FIB sections were lifted out *in situ* and attached on a copper TEM grid (without carbon membrane) by depositing Pt at the contact between the section and the grid. The section was thinned to ∼120 or 70 nm using low-beam currents (1 nA, 300 pA, 100 pA and finally, 50 pA) grazing on each side of the section. During thinning, SEM images were taken with a secondary electron detector to control the process and choose the side that had to be thinned and with an in-lens back-scattered electron detector to distinguish Fe minerals using chemical contrast. Finally, the amorphous material redeposited by the plasma during thinning was removed by scanning the section with a 5 kV ion beam at an angle of 4–7° with the section surface.

### STEM

STEM coupled with EDXS was performed on five TEM instruments to map the distribution and analyse the chemistry of organic matter and crystals at the nanoscale. We used a FEI Tecnai G2 20 (at CCM Lille) operated at 200 kV and equipped with annular dark-field and axial bright-field STEM detectors, a FEI Tecnai G2 TWIN (at Catμ Liège) operated at 200 kV and equipped with a high-angle annular dark-field detector (HAADF), a Philips CM30 (at CCM Lille) operated at 300 kV and equipped with annular dark-field, axial bright-field STEM detectors, a FEI Titan^3^ (at EMAT Antwerp) operated at 120 kV in HAADF mode and a FEI Titan^3^ (at CCM Lille) operated at 300 kV in HAADF mode. On all TEMs, we used a cryo-trap (cooled with liquid N_2_) to limit electron beam deposition of volatile contaminants. The electron beam was ca. <13 nm large for EDXS maps and STEM dark/bright-field images. The pixel size of EDXS maps was <13 nm. EDXS spectra were recorded on circular or rectangular regions as outlined in figures.

### Morphometry

Microfossils were measured using ImageJ. Average wall thickness was measured in FIB sections of *Huroniospora* as wall surface divided by wall perimeter; due to pinching and swelling of walls, this measurement is more accurate than transverse measurement of the walls. Cell diameters for filamentous morphospecies (*Gunflintia* and *Animikiea*) were measured in optical photomicrographs as the radius of the filaments. Segment lengths in *Gunflintia* were measured as detailed in Supplementary Fig. 13a.

### Selected area electron diffractions

Selected area electron diffraction (SAED) and nanobeam (low-angle-tilted convergent beam) diffraction patterns were recorded on the FEI G2 20 and the Philips CM30 TEM. At 200 and 300 kV, amorphization by radiation damage of quartz (but not Fe-bearing minerals) was fast enough to permit SAED and nanobeam diffraction on small Fe-bearing crystallites without interference of the quartz matrix (for example, double diffraction by quartz). The aperture-selected areas are outlined with a circle on SAED figures.

### Electron energy loss spectroscopy

EELS was used to investigate the redox state of Fe, the speciation of C and the environment of Si. EELS spectra were recorded in STEM mapping mode on a FEI Titan^3^ TEM (X-Ant-TEM at EMAT Antwerp) equipped with a Cs probe corrector and a Gatan Enfinium spectrometer and operated at 120 kV. The monochromator of the microscope was excited to a value of 0.6 kV, leading to an energy resolution of 200 meV with a 1 μm C1 aperture. In this Mono STEM mode, the convergence angle of the probe was 16.2 or 17.1 mrad using a limiting 30 μm C3 aperture with a beam current of 60–100 pA. We used a focused beam (for 0.1 s analyses: electron dose of ∼5 × 10^6^ electrons.Å^2^) or a beam with –12.5 μm defocus to spread the electron flux over a 130 nm-wide circle (for 30 s analyses: electron dose of 7,800 electrons per Å^2^), hence lowering fluence and avoiding irradiation damage to greenalite. EELS spectra were recorded at a camera length of 29.5 mm with a Gatan Imaging Filter entrance aperture of 2.5 mm, which corresponds to a collection angle of about 25 mrad. The minimum dispersion available (0.025 eV per pixel) allowed good visualization of the EELS features and use of dark reference correction to improve signal-to-noise ratio.

### STXM

STXM was used to perform high spatial resolution (25 nm) spectromicroscopy at the carbon K-edge (energy range 270–320 eV) to image the distributions of carbonates and organic matter and identify carbonates in X-ray absorption near-edge spectroscopy spectra. STXM analyses were performed at the Canadian Light Source beamline 10ID-1 (SM beamline) using soft X-rays generated with an elliptically polarized undulator inserted in the 2.9 GeV synchrotron storage ring. The microscope chamber was evacuated to 100 mTorr after sample insertion and back-filled with He. We used a spectral resolution of 0.8 eV between 275 and 283 eV, 0.15 eV between 283 and 295 eV, 0.5 eV between 295 and 310 eV and counting times of the order of a few milliseconds or less per pixel. Additional details on data acquisition, processing and interpretation are provided in ref. 30. Carbonate maps were obtained by subtraction of the X-ray transmission images recorded at 290.3 eV (CO_3_^2−^ absorption maximum)—285.4 (aromatic absorption maximum), and aromatic carbon maps were obtained by subtraction of X-ray transmission images at 285.4 eV (aromatics)—275 eV (pre-edge absorption ‘baseline').

### Fe minerals concentrations

The concentration of Fe minerals in Gunflint microfossils and in the surrounding quartz matrix was estimated from images of FIB sections. The cumulated surface of Fe minerals in the FIB section of thick-walled *Huroniospora* microfossils was measured in ImageJ using a threshold function selecting all Fe minerals. STEM images with minimal diffraction and sample thickness contrast are required to select Fe minerals with the threshold function: these were available for the high- (Fig. 1b) and low-Fe (Supplementary Fig. 6a) endmembers of the Fe-mineralized thick-walled *Huroniospora* and for the *G. grandis* of Fig. 2h. For *Huroniospora*, cell volumes were estimated using diameters measured on optical photomicrographs (8.7 and 7.7 μm, respectively) and assuming spherical shape. For *Gunflintia*, cell volumes were estimated using measured diameters and assuming cylindrical shape. The volume of Fe-bearing crystals was extrapolated from their area measured in FIB section as follows: (surface of Fe minerals/surface of fossil in section) × (cell volume). The density of Fe minerals (3.2 g cm^−3^) was approximated to that of a mixture of 80% greenalite and 20% siderite. Both greenalite and siderite host ∼48 weight% Fe. This led to the intracellular Fe-quota^45^ (in Fe atoms per cell) calculated as (volume of crystals) × density × 48 wt% × *N*_Avogradro_/56=2.7 × 10^12^ (Fig. 1b), 6.5 × 10^11^ (Supplementary Fig. 6a). In turn, this led to Fe concentrations of 9.6 × 10^8^ and 3.4 × 10^8^ Fe atoms per μm^3^ of cell for each *Huroniospora*, respectively, and 8.6 × 10^8^ Fe atoms per μm^3^ for *G. grandis*. Similarly, the Fe-rich nanocrystal concentration (inferred as 100% greenalite) in the quartz matrix outside microfossils analysed in all the FIB sections accounts for less than 1.5 × 10^6^ Fe atoms per μm^3^, as their small size (in contrast with larger crystals in microfossils) leads to overestimates in image analysis.

### Data availability

Repeated diffractions and EDXS spectra on greenalite and siderite nanocrystals are available from the corresponding author on request.

## Additional information

**How to cite this article:** Lepot, K. *et al*. Iron minerals within specific microfossil morphospecies of the 1.88 Ga Gunflint Formation. *Nat. Commun.* **8,** 14890 doi: 10.1038/ncomms14890 (2017).

**Publisher's note:** Springer Nature remains neutral with regard to jurisdictional claims in published maps and institutional affiliations.

## Supplementary Material

Supplementary Information Supplementary Tables, Supplementary Figures, Supplementary Discussion and Supplementary References
