# Supplementary material for: Iron minerals within specific microfossil morphospecies of the 1.88 Ga Gunflint Formation
Source: Nat Commun. 2017 Mar 23;8:14890. doi: 10.1038/ncomms14890 (PMC5376642; doi:10.1038/ncomms14890)
Supplement: Supplementary Information — Supplementary Tables, Supplementary Figures, Supplementary Discussion and Supplementary References [file ncomms14890-s1.pdf]

| **morphospecies** | **ultrastructure** | **microfossil designation** | **cell diameter** | **avg. wall thickness** | **average segment length** | | **Fe-minerals observed inside microfossils** | **Fe-minerals outside microfossils** | **Figures #** | |
|---|---|---|---|---|---|---|---|---|---|---|
| | | | | | | | | | context | nanoscale |
| | | | **µm** | **nm** | **µm** | **± 1SD** | | | | |
| **Huroniospora** | **thick-walled** | D54b#1 | 8.7 | 295 | | | greenalite, Fe-sulfides | no | S2, S3a | 1a-c, S4a |
| | | D54f | 7.7 | 151 | | | greenalite, siderite | no | S3a | S6a |
| n = 9 | | D54t2 | 8.5 | 490 | | | greenalite | no | S3a | S6b |
| | | D55t4a | 8.8 | 146 | | | greenalite, siderite | no | S3d | S6c |
| | | D55t4b | 8.0 | 155 | | | greenalite, siderite, Fe-sulfides | no | S3d | S6d |
| | | D53t1 | 12.0 | 573 | | | greenalite, siderite | no | S3c | S6f |
| | | C40-3a | 7.3 | 261 | | | greenalite, siderite | no | S3g, S3i | S6e |
| | | D55t4c | 7.9 | 113 | | | greenalite, siderite | no | S3d | S6g |
| | | D54d | 11.7 | n/a | | | Fe detected with SEM (no FIB section made to identify minerals) | no | S3a | S14a-c |
| | | | **8.4** | **average diameter of n=9 + n=10 other microfossils** | | | | | | |
| | | | | | | | | | | |
| **Huroniospora** | **thin-walled** | D54b#2 | 2.5 | 40 | | | absent | no | S2, S3a | 1a-c, S4a |
| | | C40-3b#1 | 1.5 to 2 µm | 46 | | | absent | no | S3g, S3i | S8 |
| n = 8 | | C40-3b#2 | 1.5 to 2 µm | n/a | | | absent | no | S3g, S3i | S8 |
| | | | **2.4** | average diameter of n=3 microfossils above + n=2 microfossils with diameter <4 µm | | | | | | |
| | | D54c | 7.5 | 46 | | | absent | two nanocrystals, possibly greenalite | S3a | 1d-f, S4b |
| | | D53c #1 | 8.5 | 50 | | | 3 nanocrystals, possibly greenalite | several nanocrystals, possibly greenalite | S3j | S5b |
| | | D53c #2 | 8.5 | 47 | | | absent | several nanocrystals, possibly greenalite | S3j | S5b |
| | | C40-2#1 | 7.5 | 54 | | | absent | | S3g-h | S5a |
| | | C40-2#2 | 7.3 | 56 | | | absent | | S3g-h | S5a |
| | | | **7.4** | average diameter of n=5 microfossils above + n=13 microfossils with diameter >6 µm | | | | | | |
| | | | | | | | | | | |
| **Gunflintia minuta** | **Type 1** | D54b#3 | 1.4 | | | | absent | no | S2, S3a | 1a-c, S4a |
| | | D54b#4 | 0.9 | | | | absent | no | S2, S3a | 1a-c, S4a |
| | | D54j | 1.5 | | | | absent | no | S3a | S9 |
| n = 5 | | C40-3b#3 | 1.4 | | | | absent | no | S3g, S3i | S8 |
| | | C40-3b#4 | 1.5 | | | | absent | no | S3g, S3i | S8 |
| | | | **1.4** | **average diameter of n=5 above + n=19 other microfossils** | | | | | | |
| | | | | | | | | | | |
| **Gunflintia minuta** | **Type 2** | D53b | 1.6 | | 3.3 | 0.9 | greenalite | several nanocrystals, | S3j | 2d-f, S7b |
| | | D60-4t1 | 2.1 | | 3.0 | 0.4 | greenalite, siderite | likely greenalite | S3e | S11a |
| | | E55-1t1 | 1.7 | | 3.2 | 0.6 | greenalite, siderite | (blade/plate shape) | S3d | S11a-b |
| n= 4 analyzed with FIB-STEM, others = only morphometry | | 2B | 1.8 | | 3.7 | | absent (poorly preserved, only one cell analyzed) | no | | S11c |
| | | e55-1t3b | 1.4 | | 2.8 | 0.9 | | | S3f | |
| | | e55-1t3a | 1.5 | | 2.7 | 0.6 | | | S3f | |
| | | C54-3 | 1.7 | | 3.8 | 0.9 | no mineralogical analyses | | | |
| | | D60-4t2 | 2.5 | | 3.6 | 0.3 | | | S3e | |
| | | J48-4 | 2.0 | | 3.5 | 0.7 | | | S13a | |
| | | E55-1t4 | 2.4 | | 3.3 | 0.6 | | | S3f | |
| | | | **1.9** | **average diameter of n=10 above + n=8 other microfossils** | | | | | | |
| | | | | | | | | | | |
| **Animikiea** | **Type 1** | D53a | 3.5 | | | | absent | no | S3j | S10 |
| | | H56a | 5.0 | | | | absent | no | | 2a-c, S7c |
| n = 3 | | J56b | 6.0 | | | | absent | several nanocrystals, likely greenalite | | S10 |
| | | | | | | | | | | |
| **Gunflintia grandis** | **Type 2** | E55-1t2 | 4.5 | | 5.2 | 1.1 | greenalite, siderite | no | S3d | 2g-i, S7a |
| | | E54-1a | 4.1 | | 4.3 | 1.1 | greenalite, siderite | two nanocrystals, possibly greenalite | S3b | S12 |
| n= 3 | | E54-1b | 4.5 | | 4.9 | 1.1 | Fe detected with SEM (no FIB section made to identify minerals) | no | S3b | S14d-f |

# Supplementary Table 1 | Summary of studied microfossils.

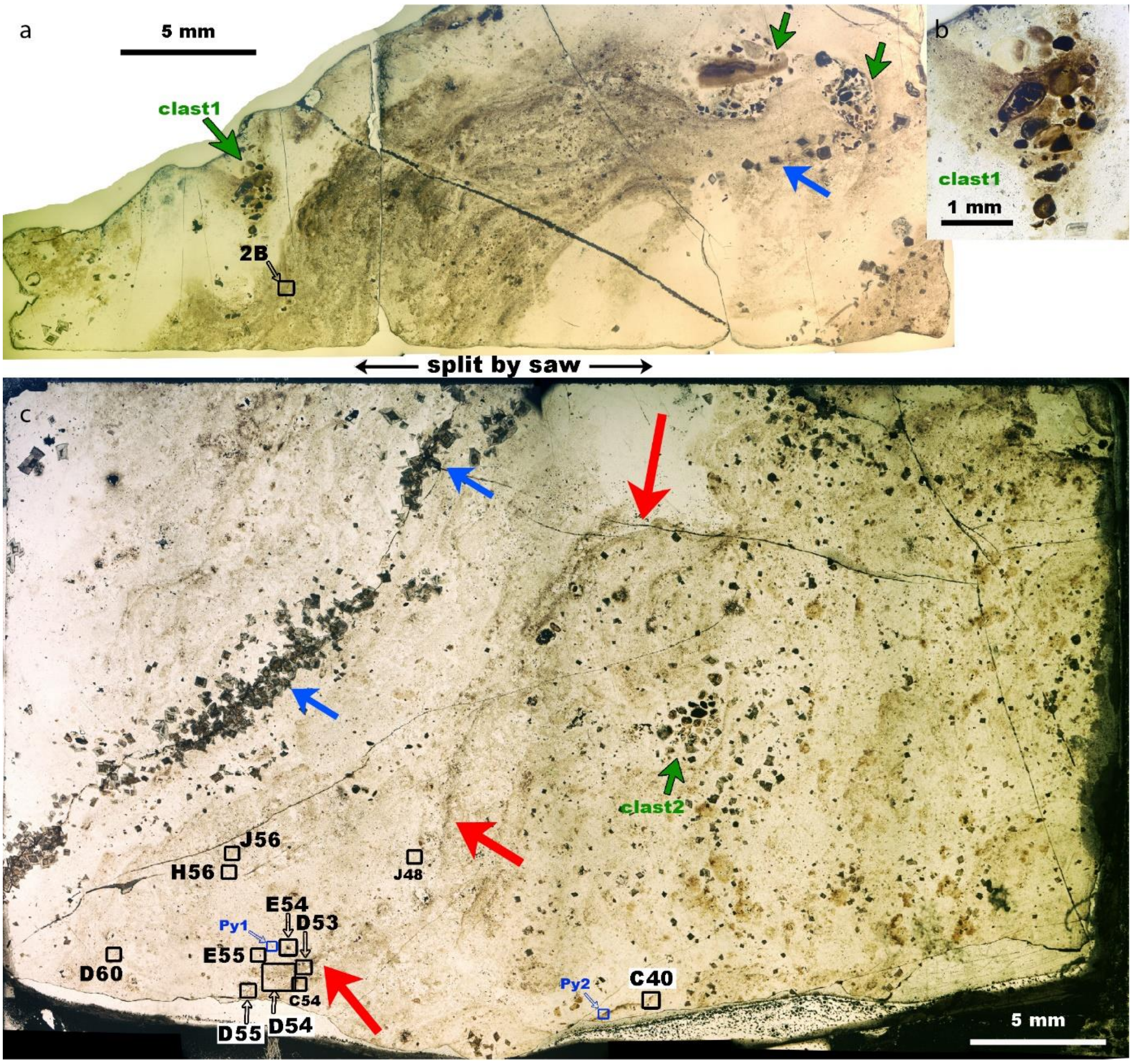


**Supplementary Figure 1 | Gunflint Formation stromatolitic chert.** Photomicrographs of two adjacent thin sections separated by a splitting gap <5 mm across. The upper (**a-b**) and lower (**c**) sections are ~100 and ~30 µm thick, respectively. Organic matter appears orange to dark brown depending on its local thickness/density in the quartz matrix. Red arrows highlight the thickest organic laminae in the lower section. Blue solid arrows highlight an ankerite-rich [(Ca,Fe,Mg)$CO_3$] vein. Pockets of rounded/coated clastic material trapped in folds of the organic laminae (green arrows in **a** and **c**, blow-up in **b** of "clast1" region arrowed in **a**) argue for shallow water deposition. D53, D54, D55, C40, E54, E55, D60, H56, J56, J48, C54, 2B, Py1 and Py2 are the studied microfossiliferous zones.

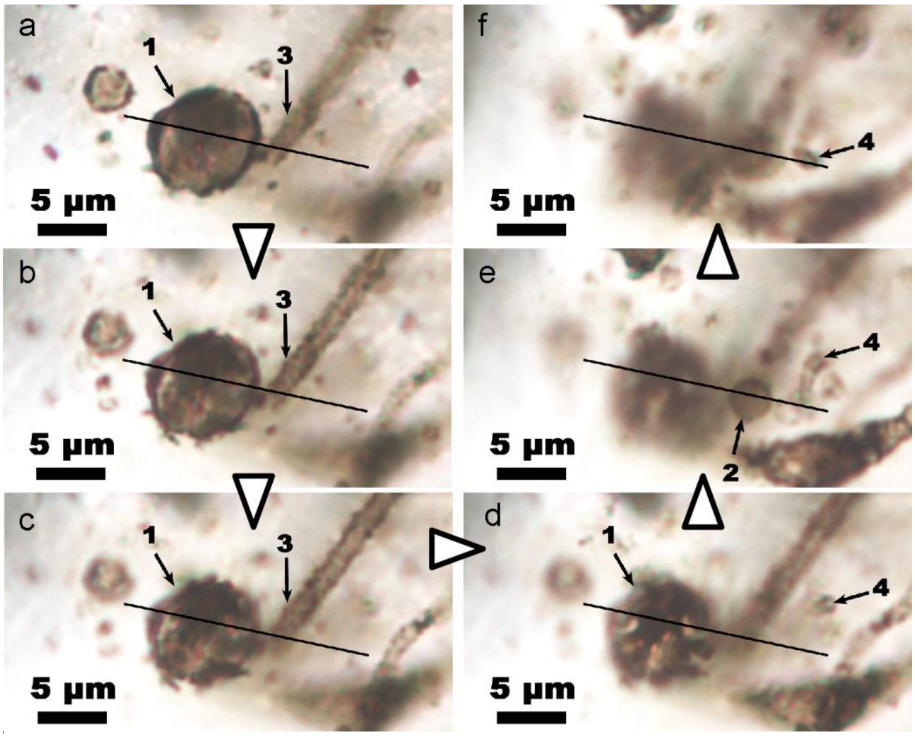


**Supplementary Figure 2 | Multiplane photomicrography of microfossil cluster D54b.** Arrows highlight studied microfossils. The six photomicrographs (**a-f**) were shot at different focal depths below the surface of the thin section (0.5, 1.5, 3, 4, 7.5 and 9.5 µm, respectively) and combined to form the multiplane image of Fig. 1a. The line highlights the FIB section location.

---

The next six pages display:

**Supplementary Figure 3 | Contextual photomicrographs of studied microfossiliferous zones. a,** D54. **b,** E54-1. **c,** D53t1. **d,** D55t4. **e,** D60-4. **f,** E55. **g-i,** C40 and Py2. **j,** D53. Lines and arrows locate microfossils sectioned with FIB. In **d**, arrows show the proximity of thin-walled *Huroniospora* (red), thick-walled *Huroniospora* (green) and type 1 *Gunflintia minuta* (blue) with thin sheaths.

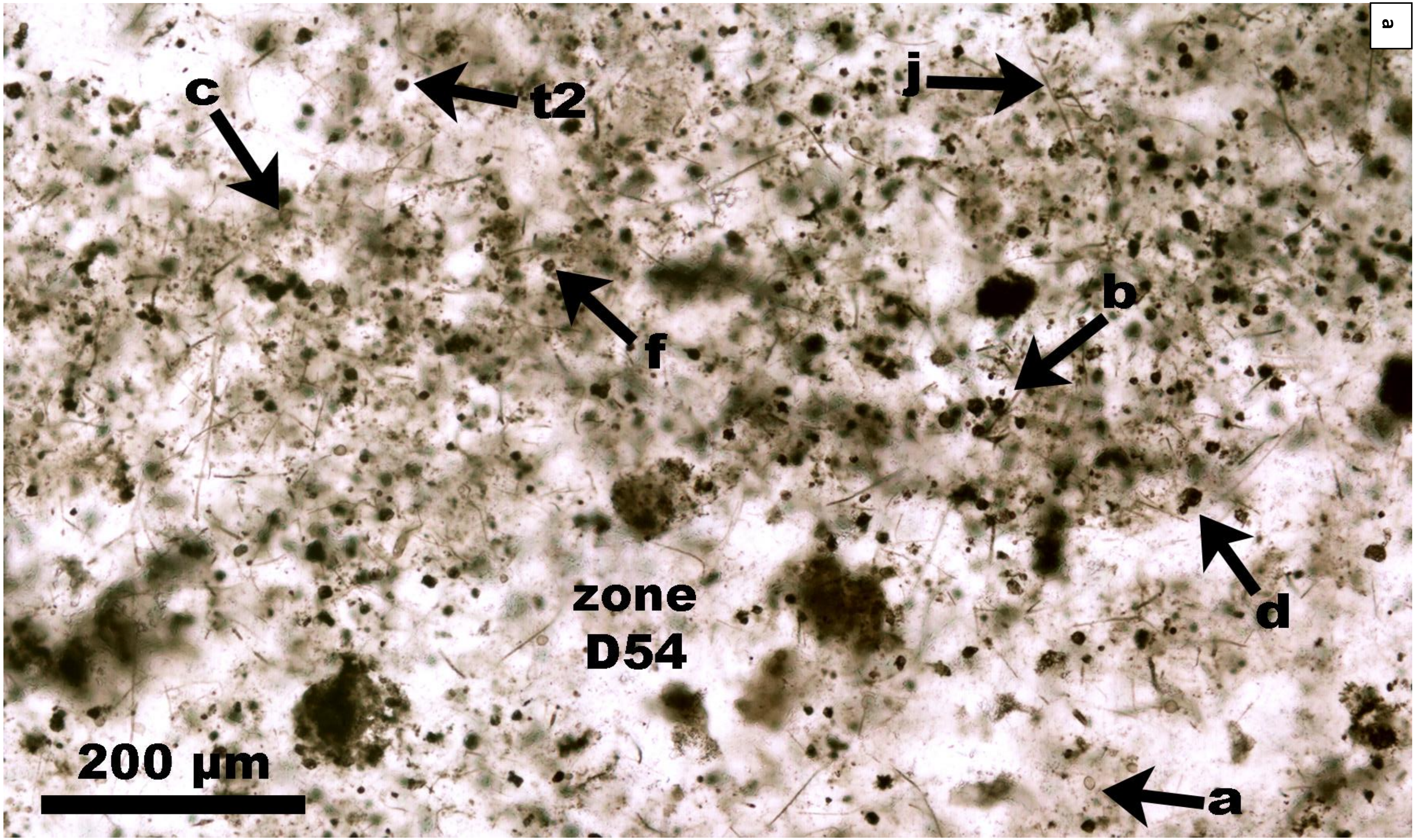
a
c
t2
j
b
f
d
zone
D54
200 µm
a

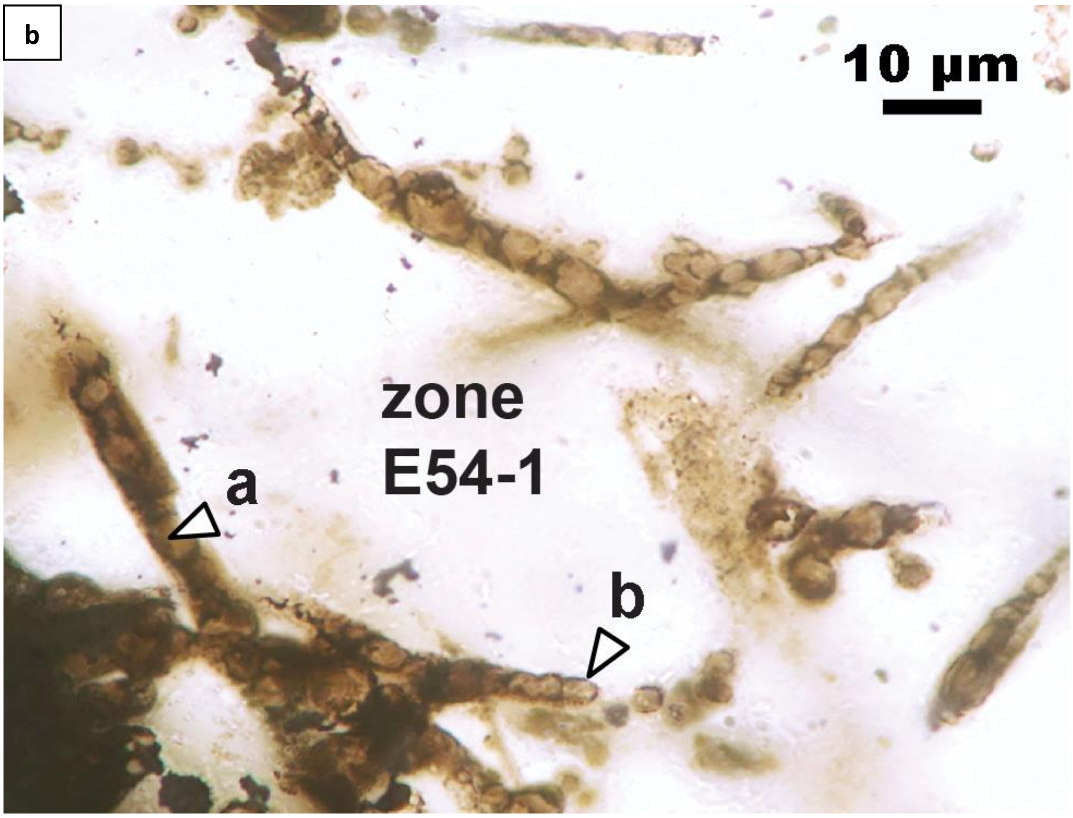
b
10 µm
zone
E54-1
a
b

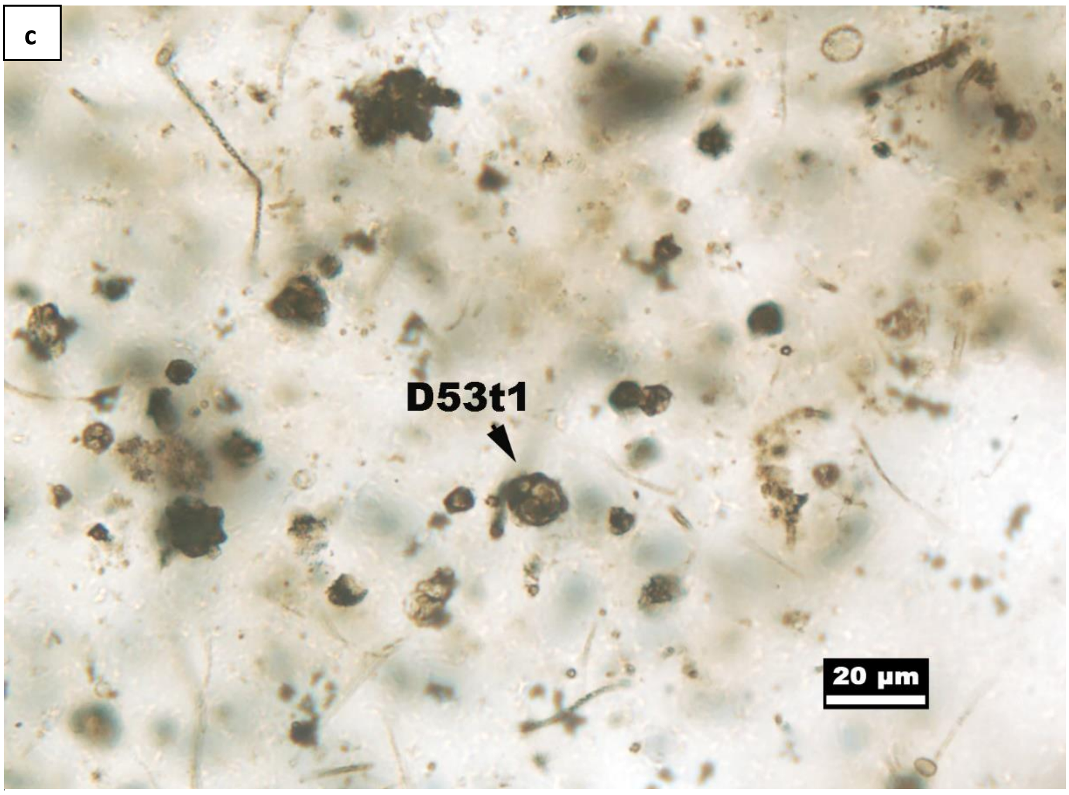
c
D53t1
20 µm

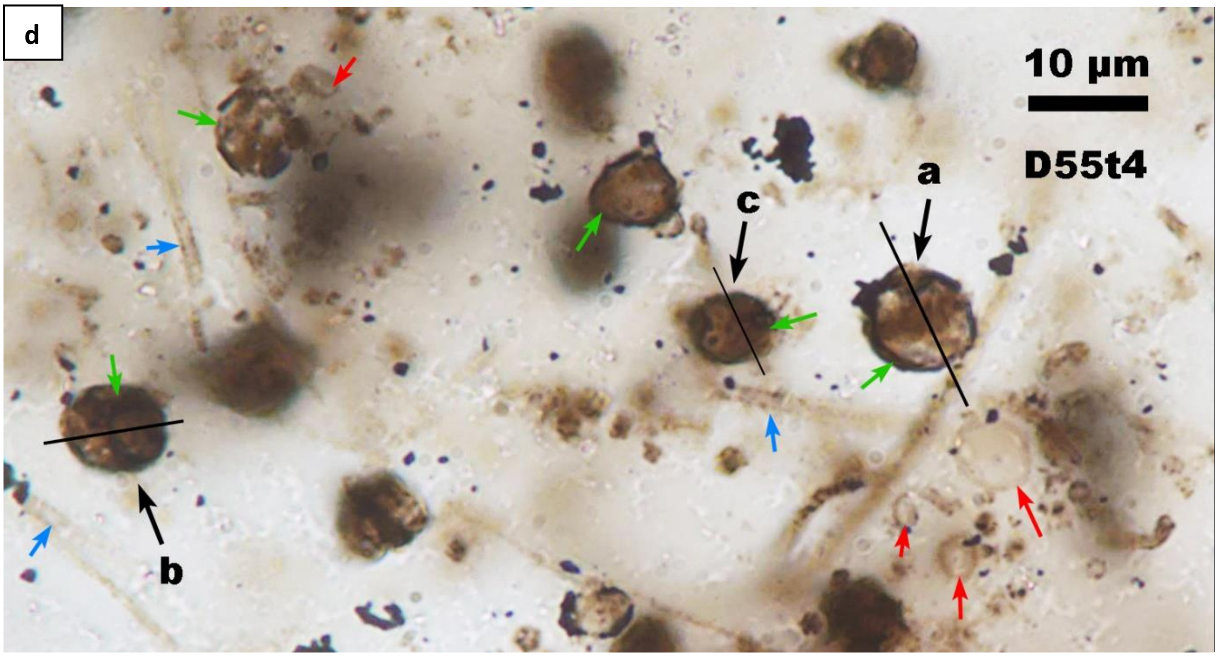
d
10 µm
D55t4
a
b
c

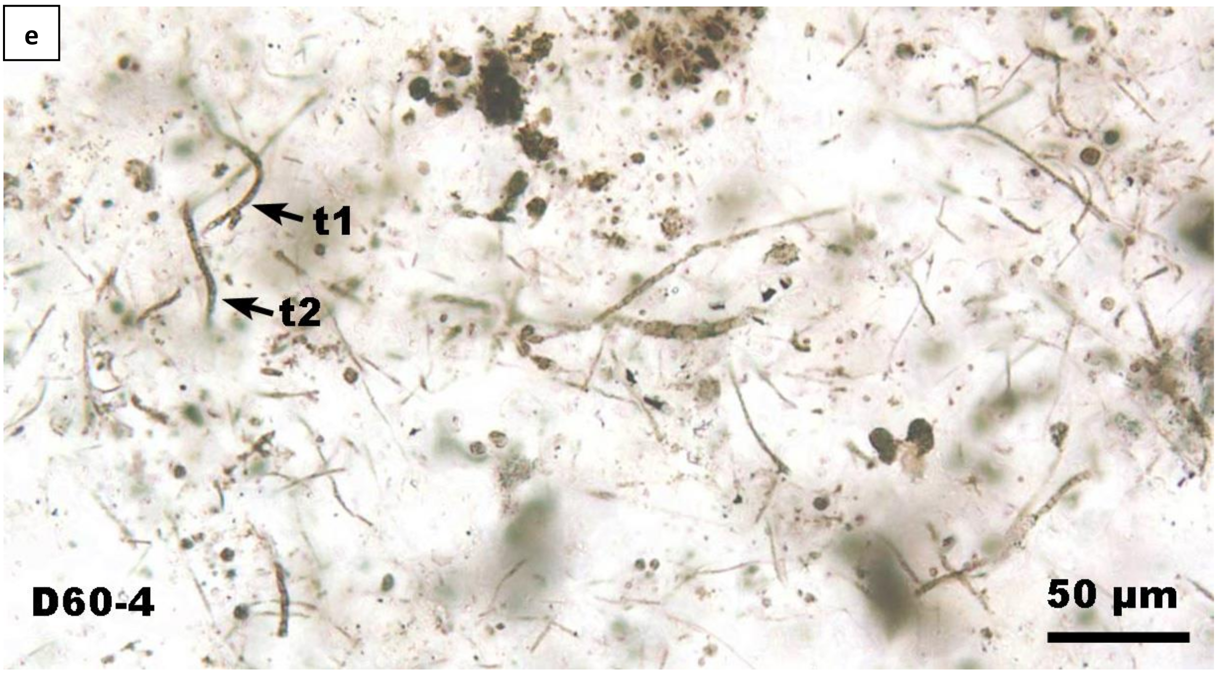
e
t1
t2
D60-4
50 µm

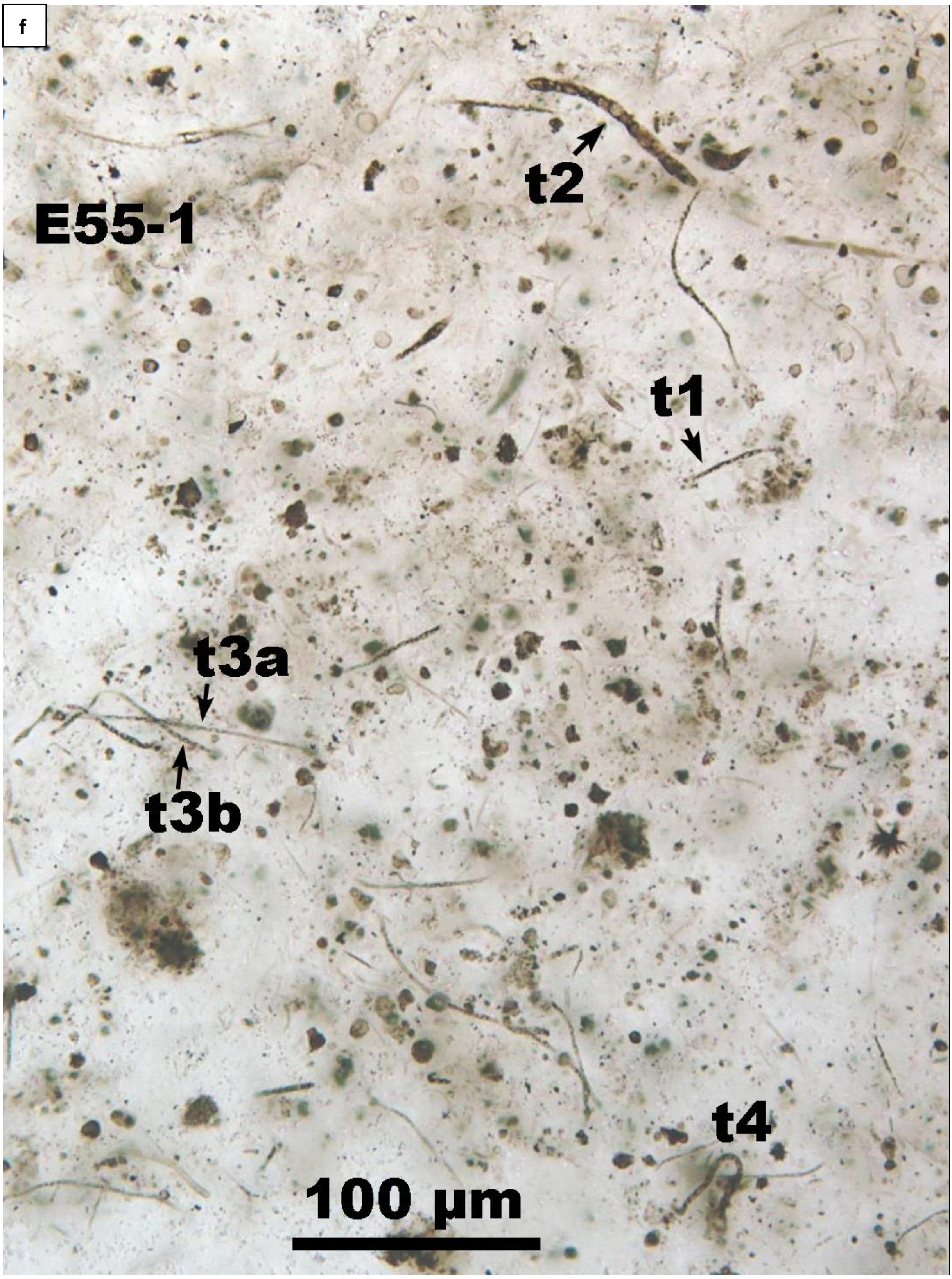
f
E55-1
t2
t1
t3a
t3b
t4
100 µm

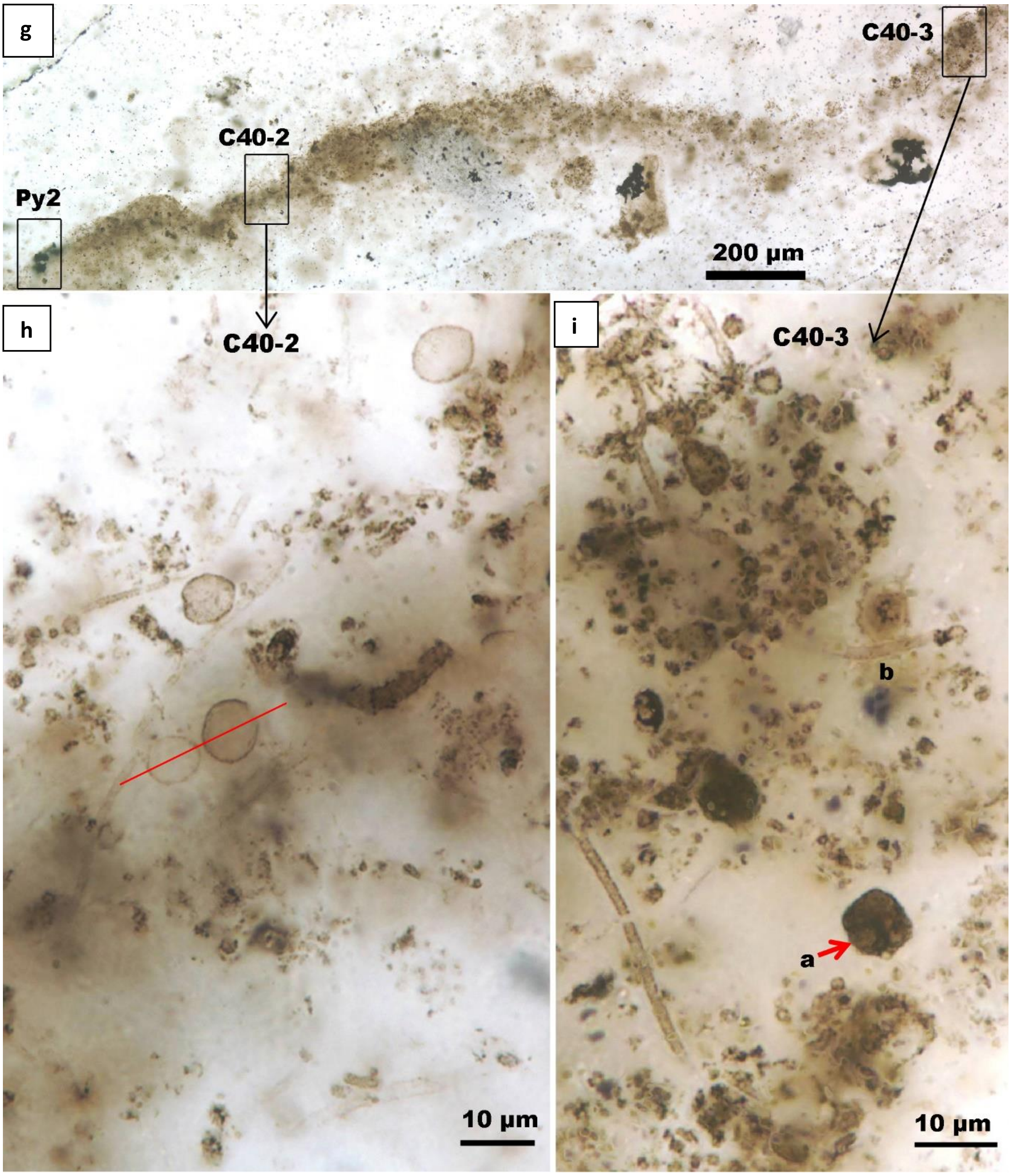

g
C40-3
C40-2
Py2
200 µm
h
C40-2
i
C40-3
b
a
10 µm
10 µm

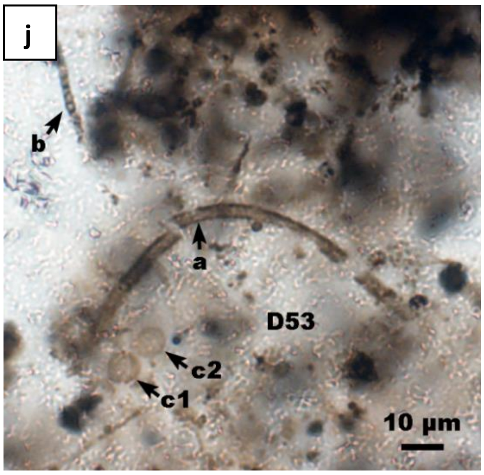


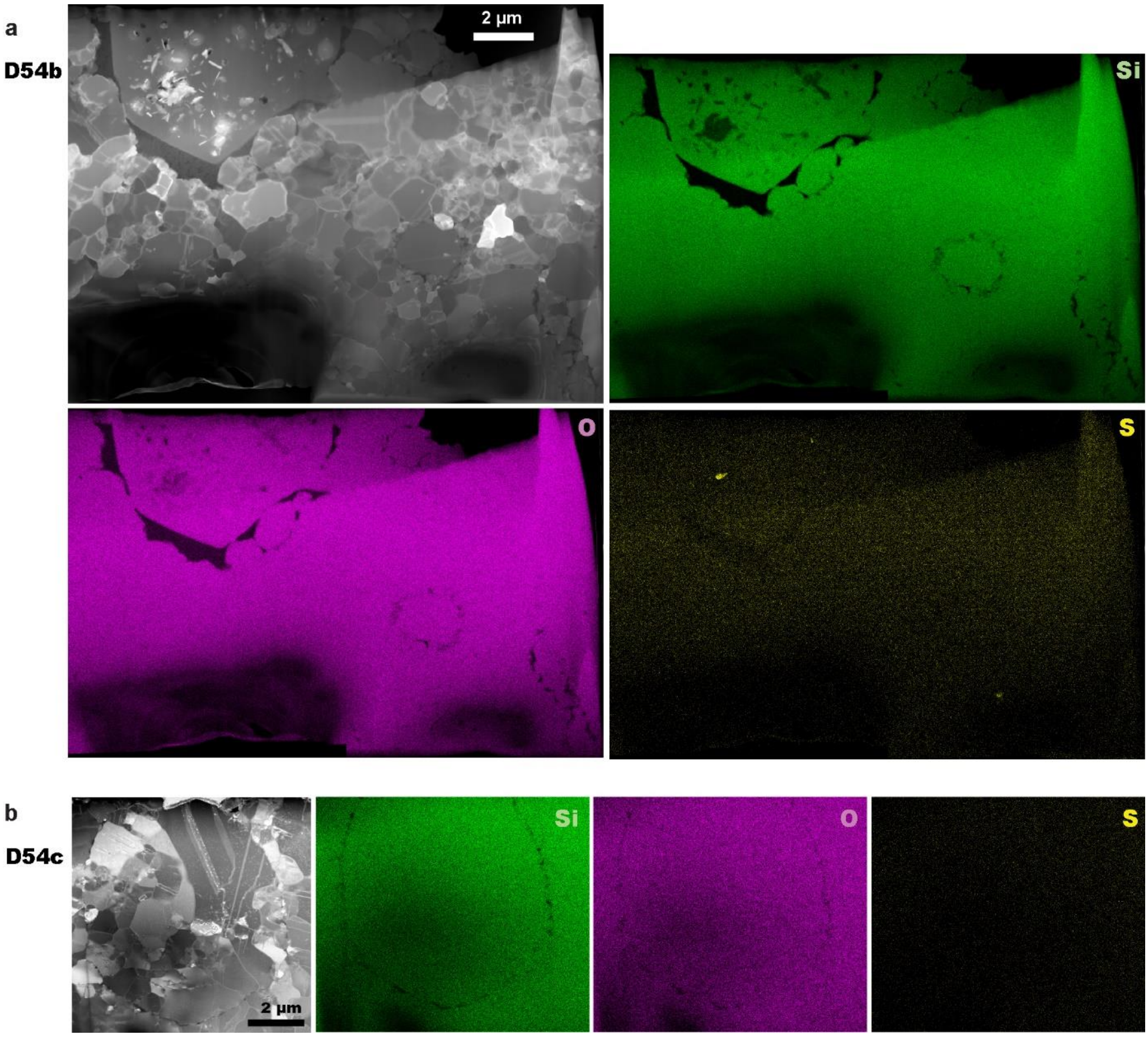


**Supplementary Figure 4 | Additional elemental STEM images and STEM-EDXS maps of microfossiliferous zones D54b (a) and D54c (b) shown in Fig. 1.** Note the few sulphide nanocrystals in the S map of **a**, which are detailed in Fig. 4. Greenalite crystals show lower intensities in Si and O maps.

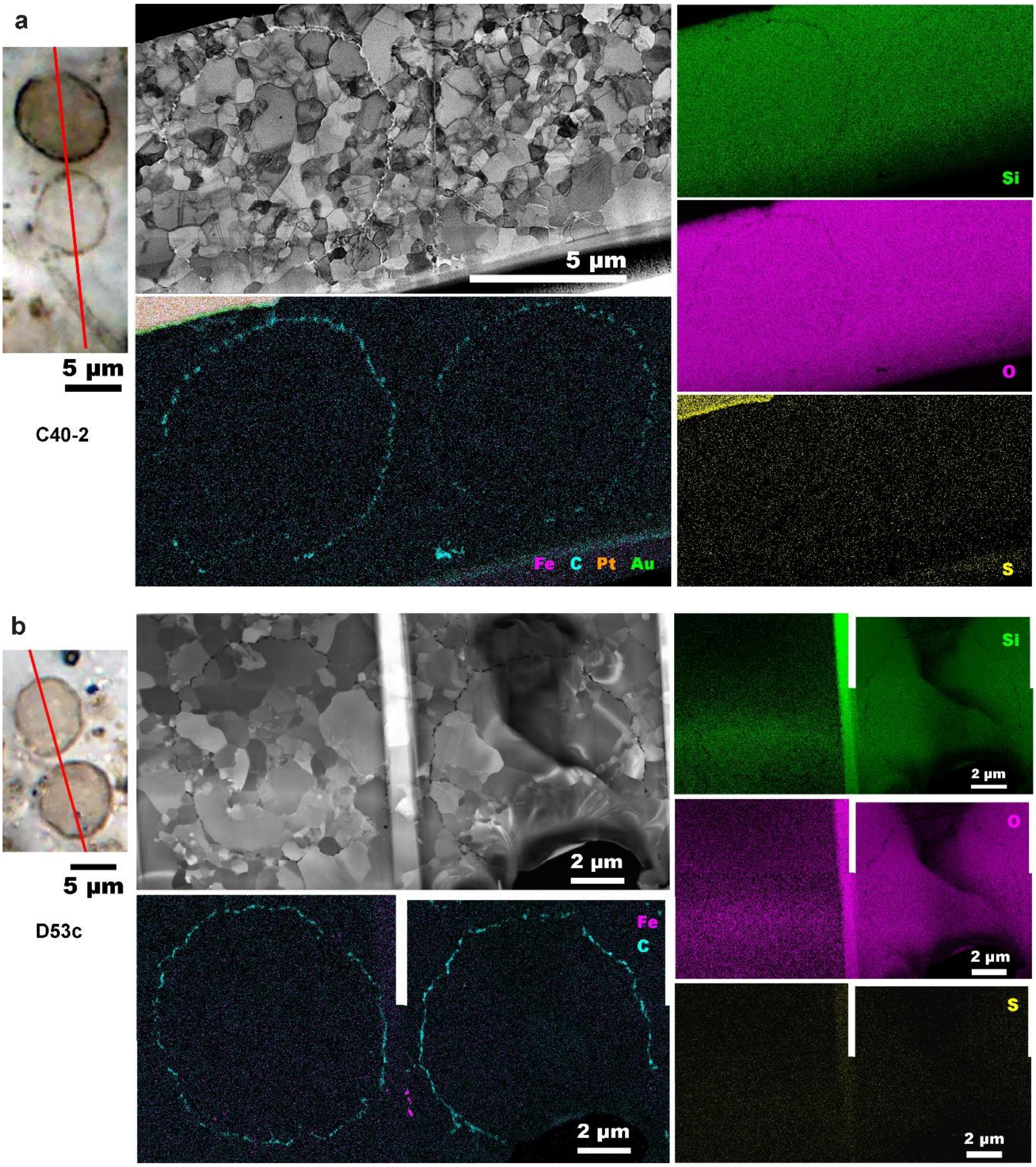


**Supplementary Figure 5 | Thin-walled *Huroniospora* microfossils. a,** C40-2. **b,** D53c. Each panel sequentially shows a multiplane photomicrograph, a STEM bright- (**a**) or dark- (**b**) field image of the FIB section cut along the red line (quartz appears in grey, organic matter in white in **a**, black in **b**), and STEM EDXS elemental maps. Platinum interferes with sulfur X-ray bands, creating an artefact of S-detection at the periphery of the FIB section on the S maps. The bright linear blade at the center of the STEM image in **b** is a thicker region of the FIB section, which was thinned in two steps (one thinning panel for each microfossil, resulting in a thicker bar at the center of the FIB section); variations in FIB thickness result in variations in intensities in all elemental maps. The white band at the top of maps in **b** indicate lack of mapping data (this map results of the stitching of 4 adjacent maps). Only scarce nanoscale Fe-bearing minerals (likely greenalite) occur outside microfossils in D53c.

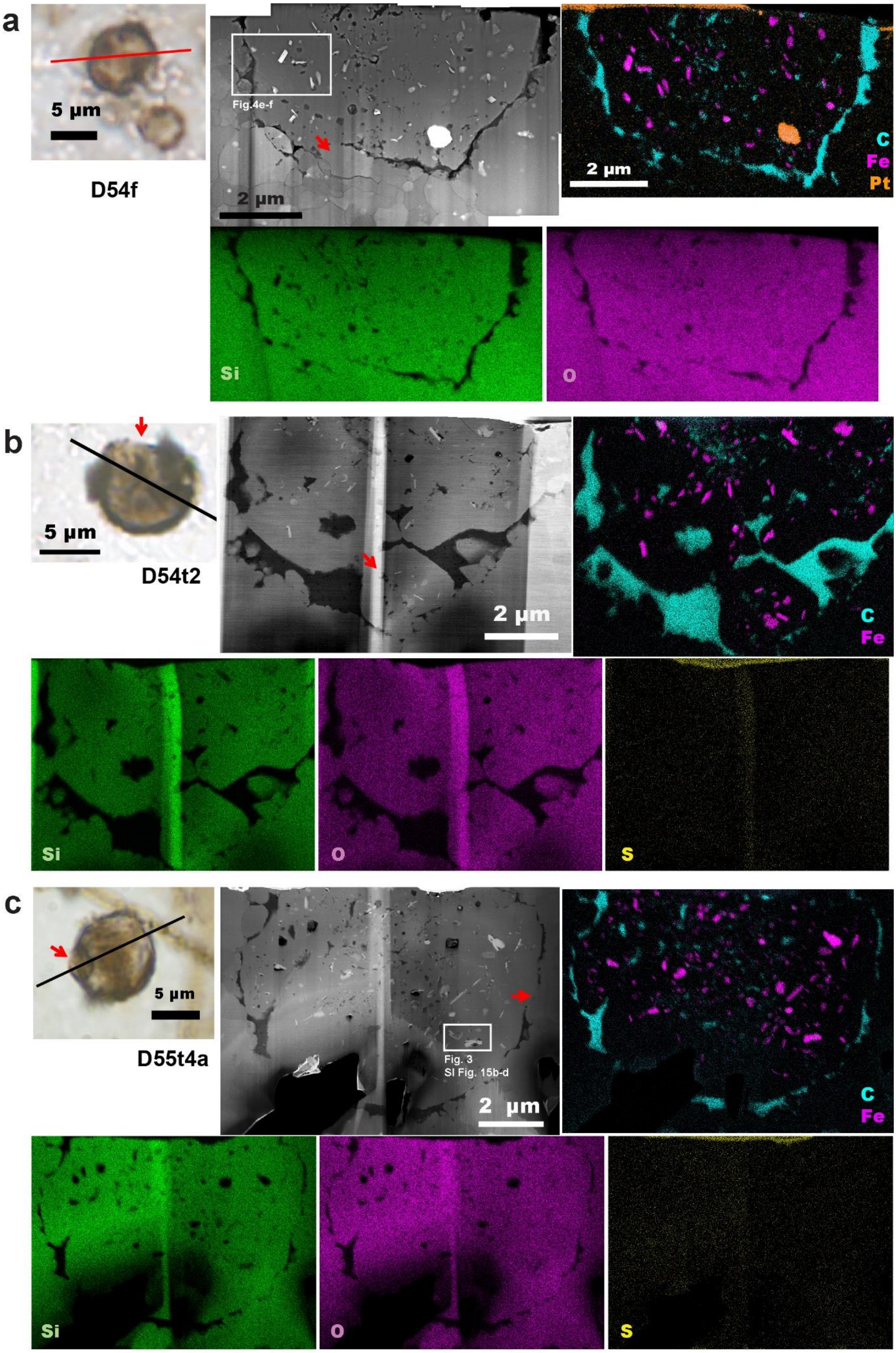


continued next page

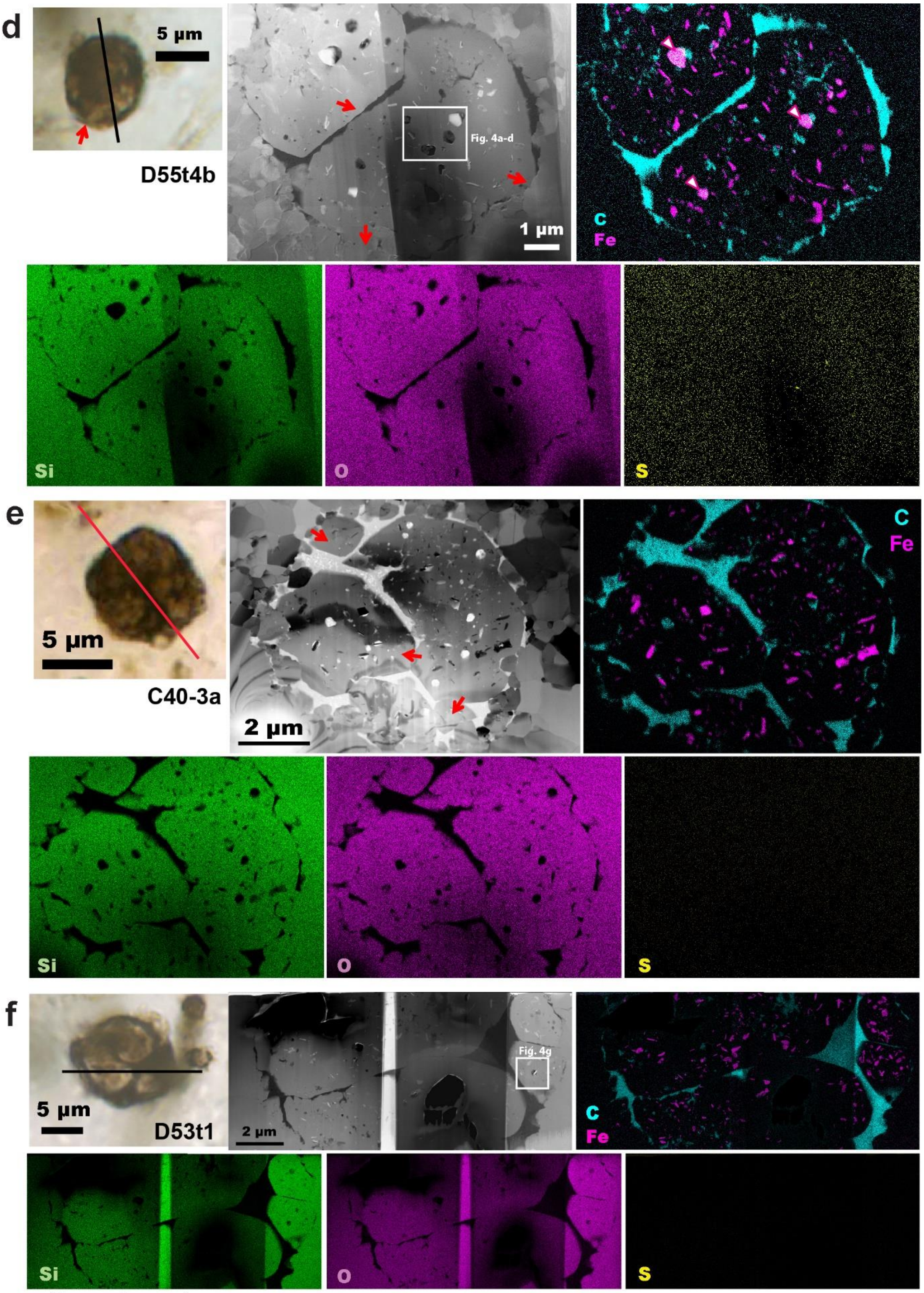


continued next page

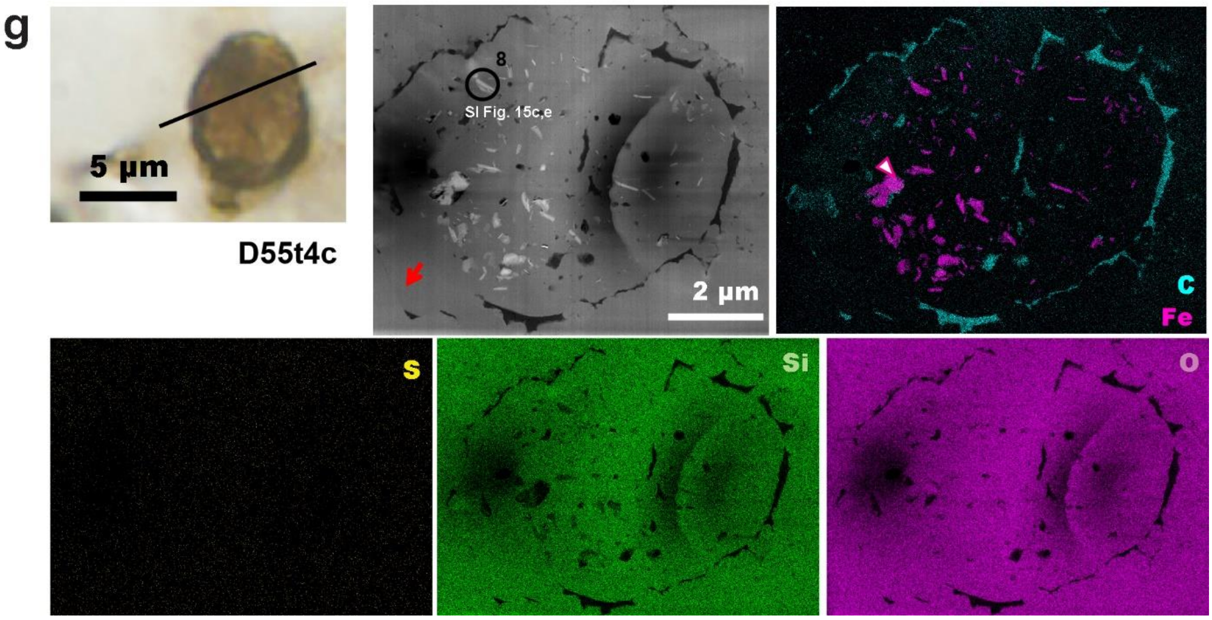


**Supplementary Figure 6 | Thick-walled *Huroniospora* microfossils of** zones D54f (**a**), D54t2 (**b**), D55t4a (**c**), D55t4b (**d**), C40-3 (**e**), D53t1 (**f**), and D55t4c (**g**). Each panel sequentially shows a multiplane photomicrograph, a STEM bright- (**e**) or dark- (**a-d**, **f-g**) field image of the FIB section cut along the red or black line, and STEM EDXS elemental maps. Fe-bearing minerals occur inside the cells, but not outside. Arrowheads highlight correlated C and Fe in the largest crystals of siderite. The discontinuities in the cell walls of these cells can be explained by the growth of quartz crystals through the cell walls (arrows). We interpret these discontinuities as *post-mortem* rather than primary features of the cells such as wall reticulation, budding or opening of cells. Note that the platinum grain observed on the FIB section in **a** is a particle that was redeposited after the tearing of the Pt-coating in the top-right part in the FIB section.

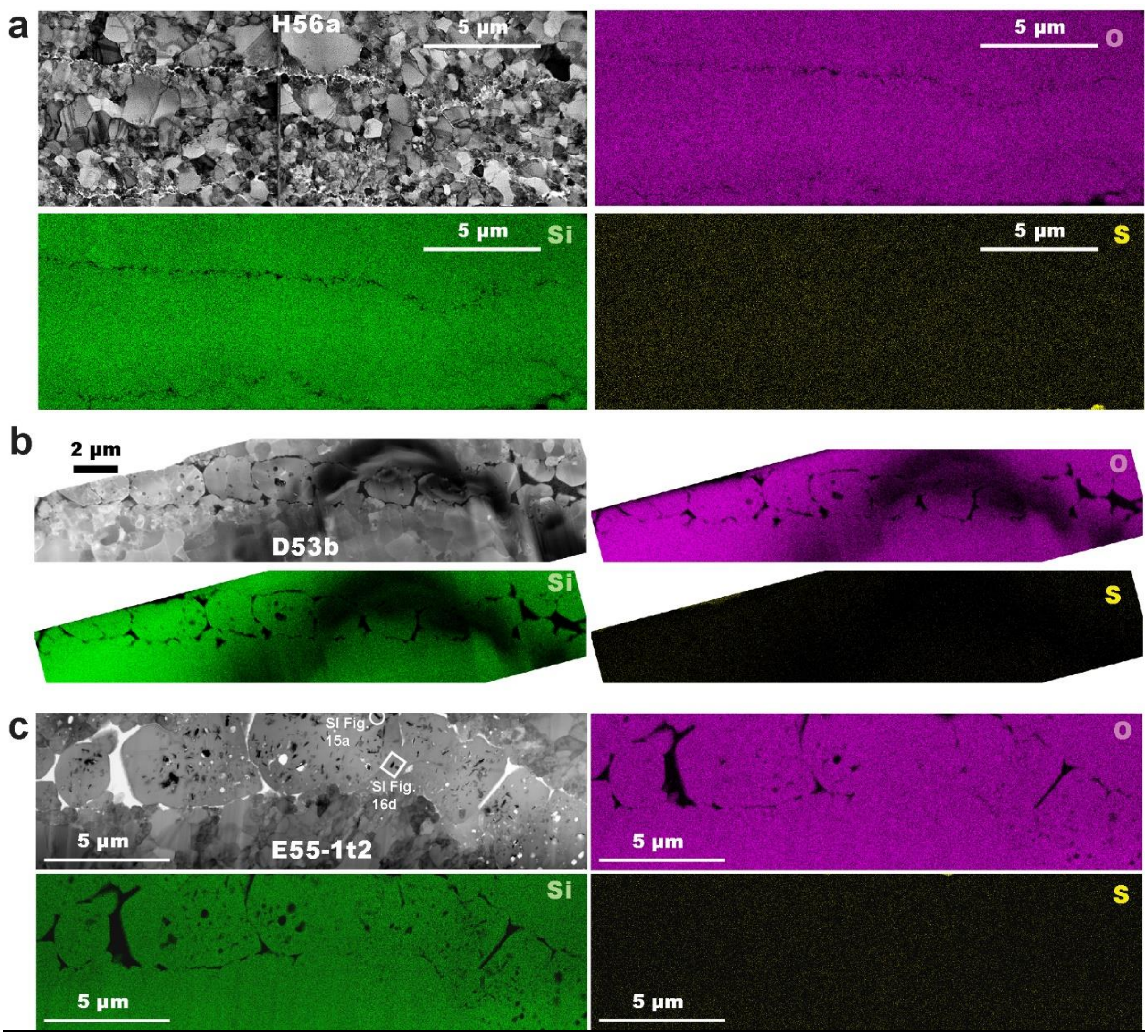


**Supplementary Figure 7 | Additional elemental STEM images and STEM-EDXS maps of microfossiliferous zones H56a (a), D53b (b), and E55-1t2 (c) shown in Fig. 2.** Greenalite crystals show lower intensities in Si and O maps. Sulphides are absent in these microfossils.

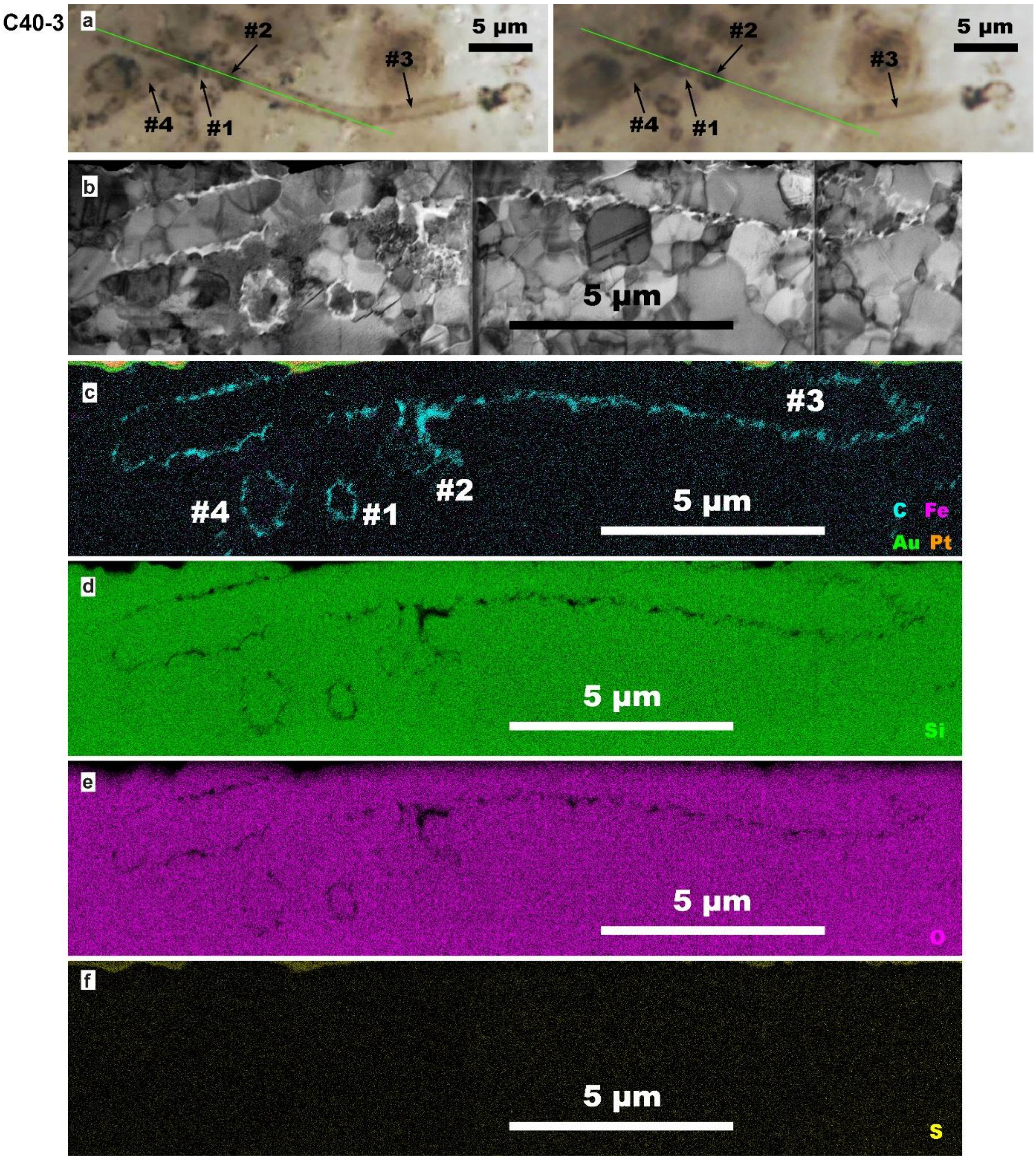


**Supplementary Figure 8 | Microfossils of region C40-3**. **a,** multiplane photomicrograph (left) and single plane photomicrograph recorded at 2.5 µm depth in the thin section (right). Microfossils #1 and #2 are small thin-walled *Huroniospora*, and microfossils #3 and #4 are Type 1 *Gunflintia Minuta*. **b,** STEM bright-field image of the FIB section cut along the green line in **a**. **c-f,** STEM-EDXS elemental maps of **b**. Fe-minerals are absent in/near these microfossils.

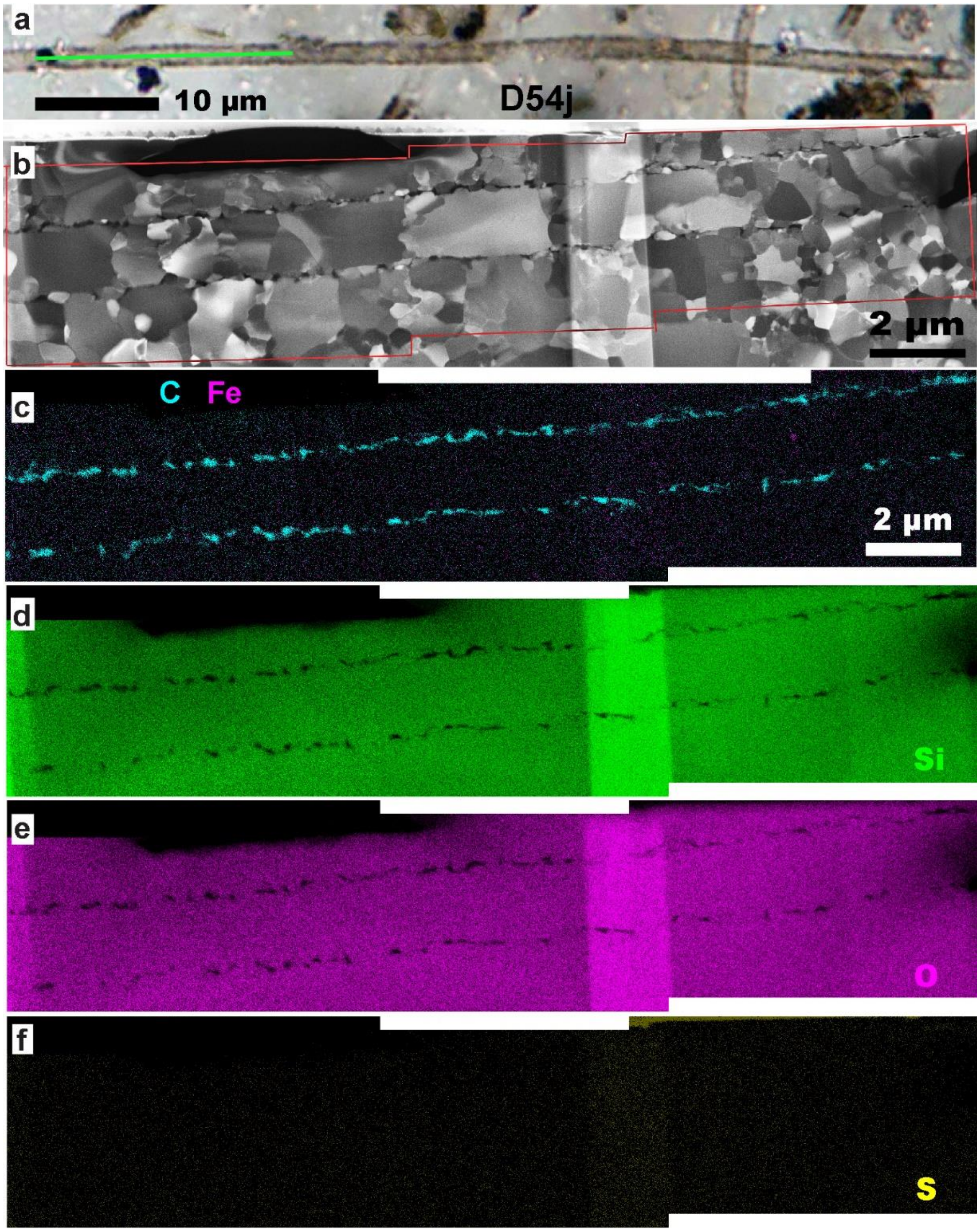


**Supplementary Figure 9 | Type 1 *Gunflintia Minuta* D54j**. **a,** multiplane photomicrograph **b,** STEM dark-field image of the FIB section cut along the green line in **a**. **c-f,** STEM-EDXS elemental maps of **b**. Fe-minerals are absent in/near this microfossil.

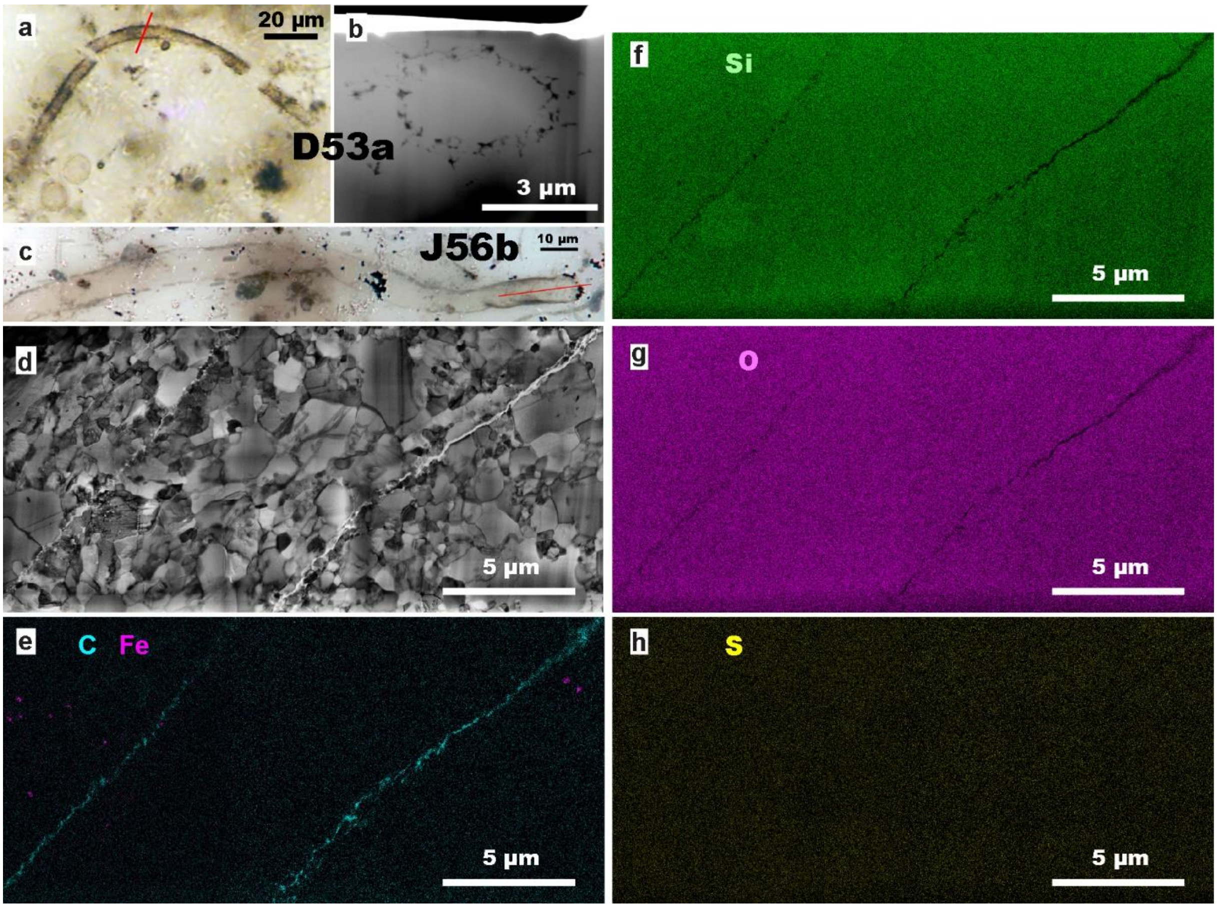


**Supplementary Figure 10 | *Animikiea* microfossils of zones D53a (a-b) and J56b (c-h)**. **a** and **c,** Multiplane photomicrographs of the broad sheathed filamentous *Animikiea*. **b,** Backscattered electron FIB-SEM image of the FIB section cut along the red line in **a**. Fe-bearing minerals are not detected in/near this *Animikiea*. **d,** STEM bright-field image of the FIB section cut along the red line in **c**. **e-h,** STEM EDXS maps of **d**. Several Fe-bearing nanocrystals occur scattered outside, but not inside this *Animikiea*.

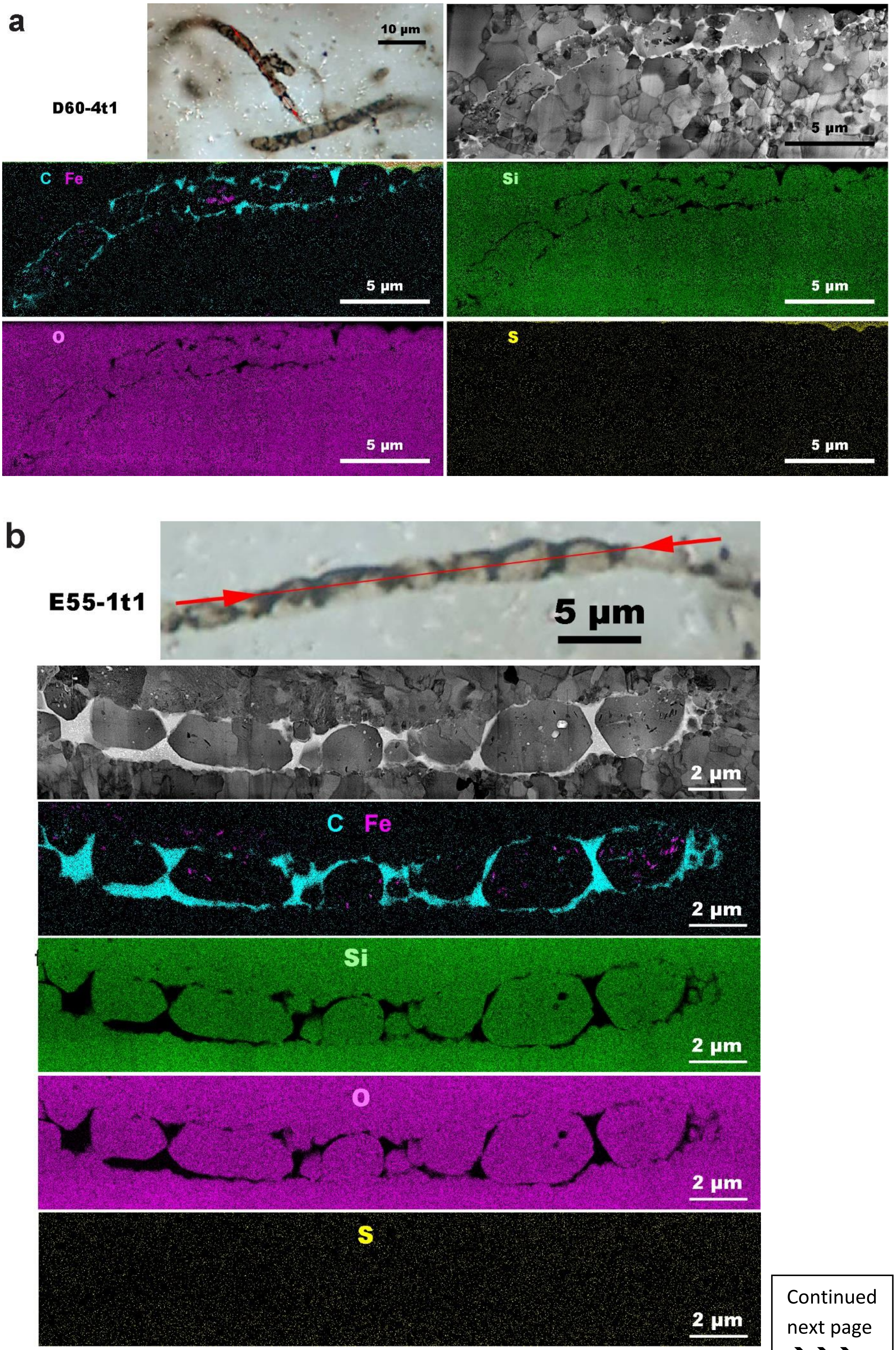


Continued next page →→→

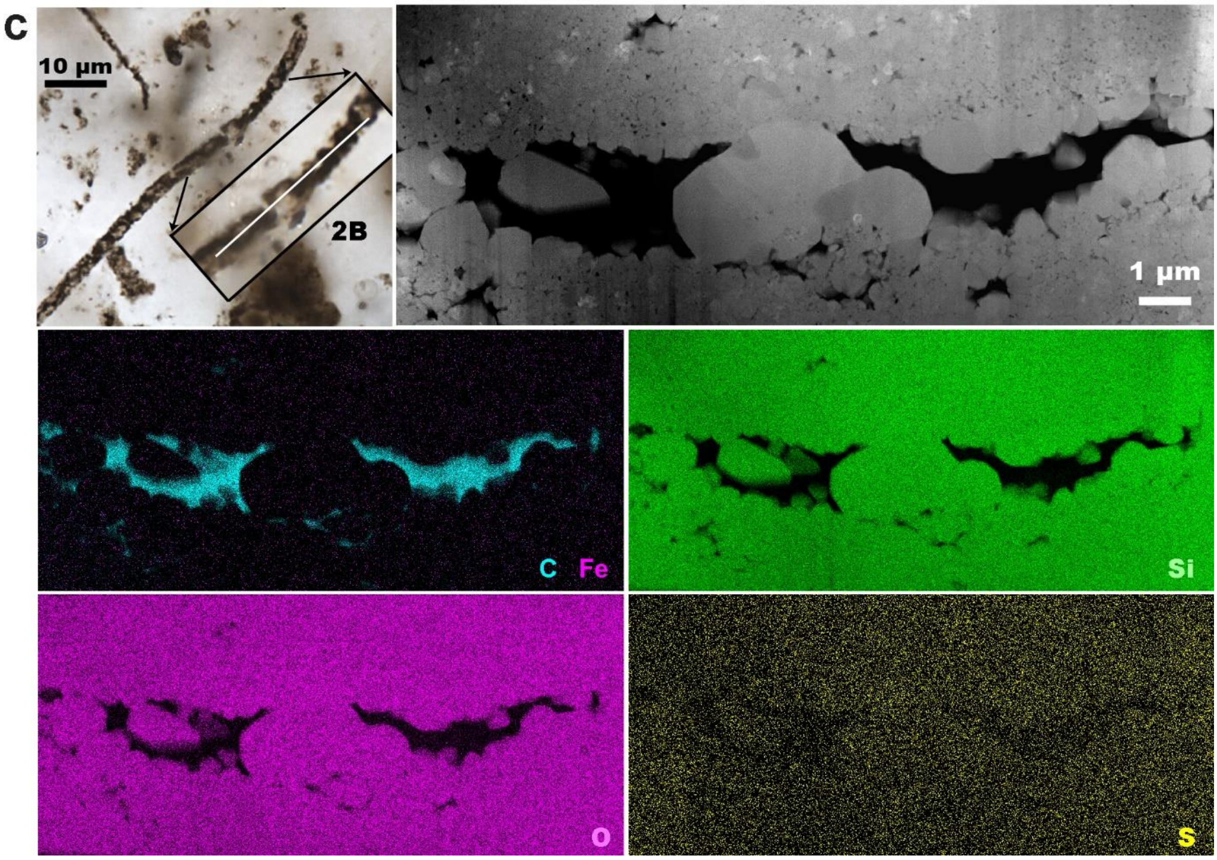


**Supplementary Figure 11 | Type 2 *Gunflintia minuta*. a,** microfossil D60-4t1. **b**, microfossil E55-1t1. **c**, microfossil 2B. Each panel (**a-c**) sequentially shows a multiplane photomicrograph, a STEM bright- (**a-b**) or dark- (**c**) field image of the FIB section cut along the red or white line, and STEM EDXS elemental maps. **a-b** show chains of cell-like quartz crystals coated by organic matter. Fe-minerals occur dominantly in these cell-like quartz crystals. **c**, in this section of a more degraded Type 2 *Gunflintia minuta*, only one cell-shaped quartz crystal is observed, while the rest of the filament consist in a >1 µm thick wisp of organic matter. Such a wisp in degraded Type 2 *Gunflintia minuta* is too thick to be explained by the collapsing of thin Type 1 sheath seen in Supplementary Figs. 8-10 and in Fig. 2a-c.

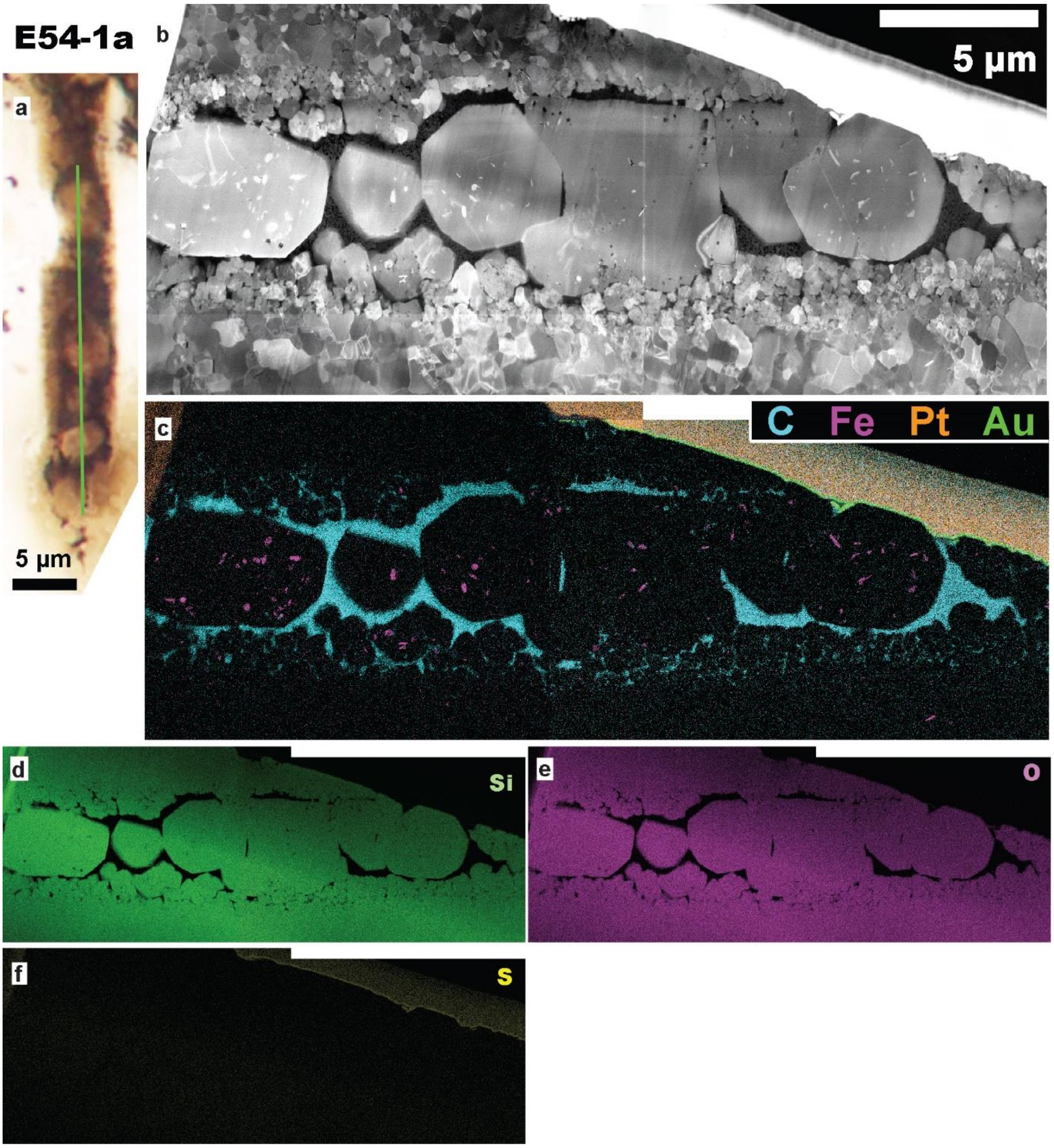


**Supplementary Figure 12 | *Gunflintia Grandis* microfossil of** zone E54-1a. **a,** Multiplane photomicrograph. **b,** STEM dark-field image of the FIB section cut along the green line in a. **c-f,** STEM EDXS maps of **b**. Fe-bearing nanocrystals occur in cell-like quartz crystals forming a chain in this filamentous microfossil. Sulphides are absent in this microfossil (the S-signal outside of the FIB section in **f** originates from Pt-interference).

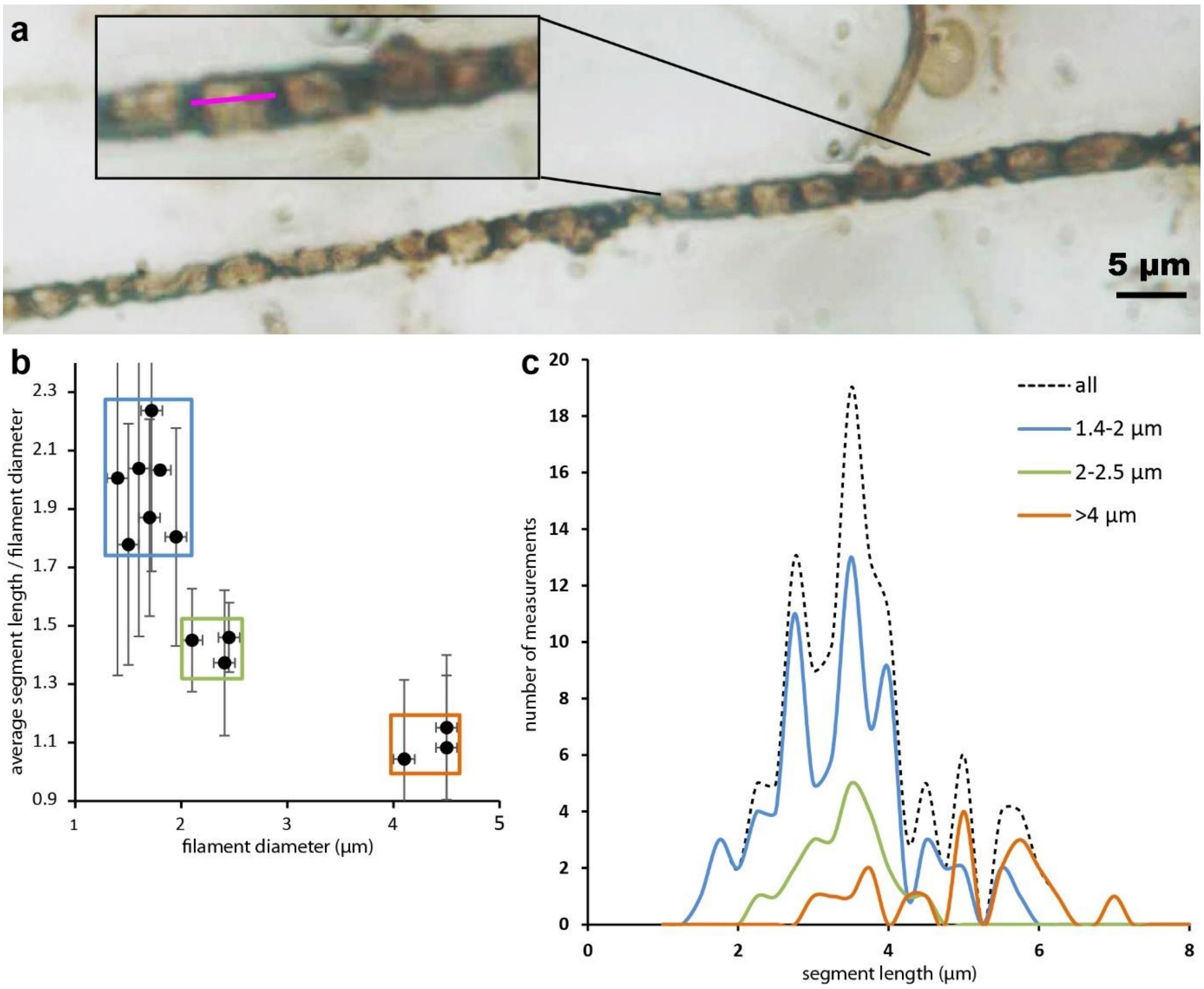


**Supplementary Figure 13 | Morphometry of segmented (Type 2) *Gunflintia* filaments. a,** multiplane photomicrograph of *Gunflintia minuta* from region J48-4. The length of cell-shaped domains, bound by filament-perpendicular organic segments was measured using imageJ as the maximum length of the quartz crystals in FIB sections, or more commonly in photomicrographs from the middle of each organic segment to the next (e.g. pink line in the zoom inset). **b**, Ratio of the average length of segment to filament diameter in each microfossil, reported against filament diameter. Vertical error bars represents one standard deviation, and horizontal error represents +/- 100 nm error linked with measurement reproducibility. The length/width ratio (elongation) parameter has been chosen because it can relate to cellular ultrastructure and filament stiffness parameters[1]. Filaments can be sorted as three groups: *Gunflintia minuta* with 1.4<diameter<2µm and 1.7<average length/diameter<2.3 (blue), *Gunflintia minuta* with 2<diameter<2.5µm and 1.3<average length/diameter<1.5 (green), and *Gunflintia grandis* with diameter>4µm and 1<average length/diameter<1.2 (orange). **c**, Histogram of all segment length measurements. The narrower Type 2 *G. minutia* (blue) show a distribution of segment lengths with sharp maxima at 2.75, 3.5 and 4 µm. The slightly broader Type 2 G. minutia (green) are mainly characterized by a maximum at 3.5 µm with shoulders at lower lengths. *G. grandis* (orange) display a much higher variability in segment length.

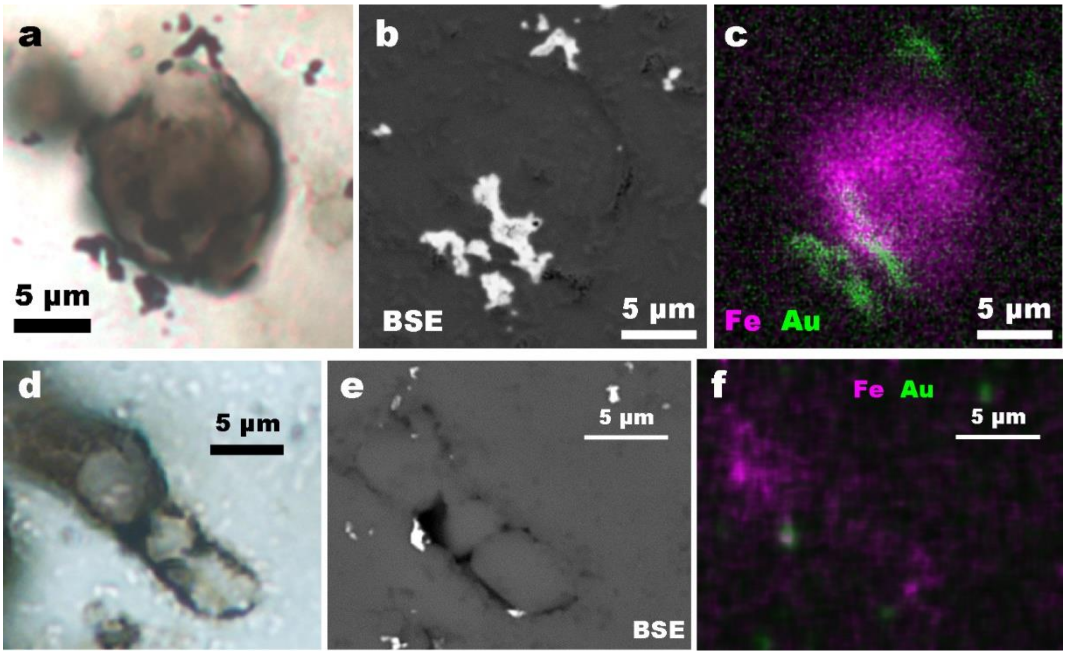


**Supplementary Figure 14 | Additional microfossils analyzed with SEM.** (**a-c**) Thick-walled *Huroniospora* of region D54d. (**d-f**) *Gunflintia Grandis* of region E54-1b. **a** and **d**, single plane photomicrographs. **b** and **e**, SEM backscattered electrons images. Quartz appears in grey. Remains of a previous gold coating (now mostly removed through re-polishing of the thin section) appear in white. **c** and **f**, SEM EDXS maps recorded at high electron beam power (30kV and spotsize#6 for ca. 1 hour) in order to detect Fe-bearing crystallites in the first micrometer inside the outcropping quartz grains (this is necessary as outcropping organic matter and Fe-minerals were largely removed through polishing). In contrast to the more energetic X-rays generated by iron, carbon within the quartz crystals cannot be mapped using this technique because of X-ray reabsorption by the matrix.

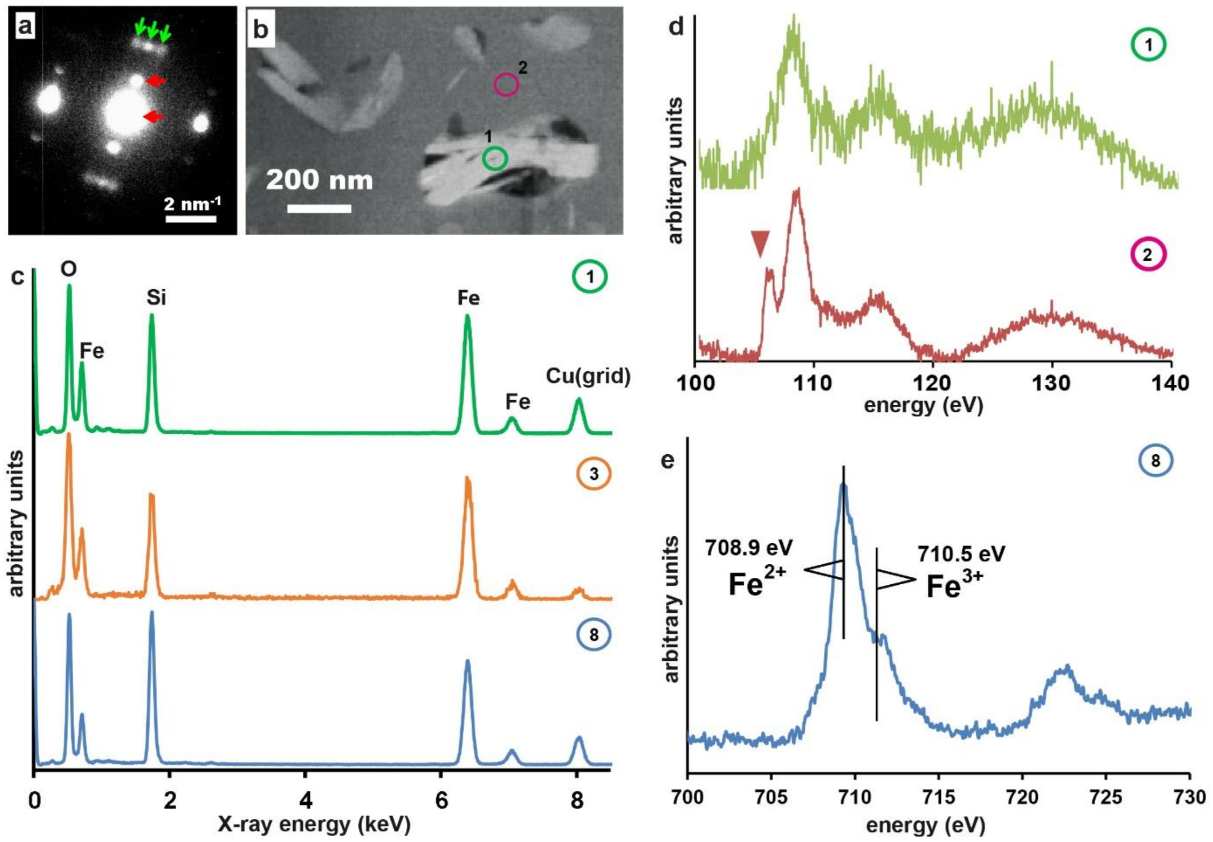


**Supplementary Figure 15 | Identification of greenalite**. **a**, SAED of greenalite crystal in *Gunflintia Grandis* E55-1t2 (circled in Supplementary Fig. 7c). Arrows indicate lattice spacing diagnostic of greenalite[2]: 7.2 Å (0,0,1) planes (red), and 23 Å superlattice (green). **b**. STEM dark-field image of greenalite. The crystals in the green circle traverse the FIB section, allowing analyses without quartz interference. **c,** STEM EDXS spectra of greenalite in the green circle in **b** (1: same zone as in Fig. 3a), of the circle (3) in Fig. 4h and of the circle (8) in Supplementary Fig. 6g. **d,** Si $L_{2,3}$ edge EELS spectra distinguishing the silicon structural arrangements of greenalite and quartz in circled regions in **b**. **e,** Fe $L_{2,3}$ edge EELS spectra of greenalite crystals (8 in Supplementary Fig. 6g) showing bands of $Fe^{2+}$ and $Fe^{3+}$: their relative heights[3] are indicative of ca. 4.5% $Fe^{3+}/Fe_{total}$. However, such a small amount of $Fe^{3+}$ might be explained by TEM-induced oxidation of $Fe^{2+}$ in greenalite in a superficial layer (<10 nm-thick) that was made amorphous during FIB preparation[4].

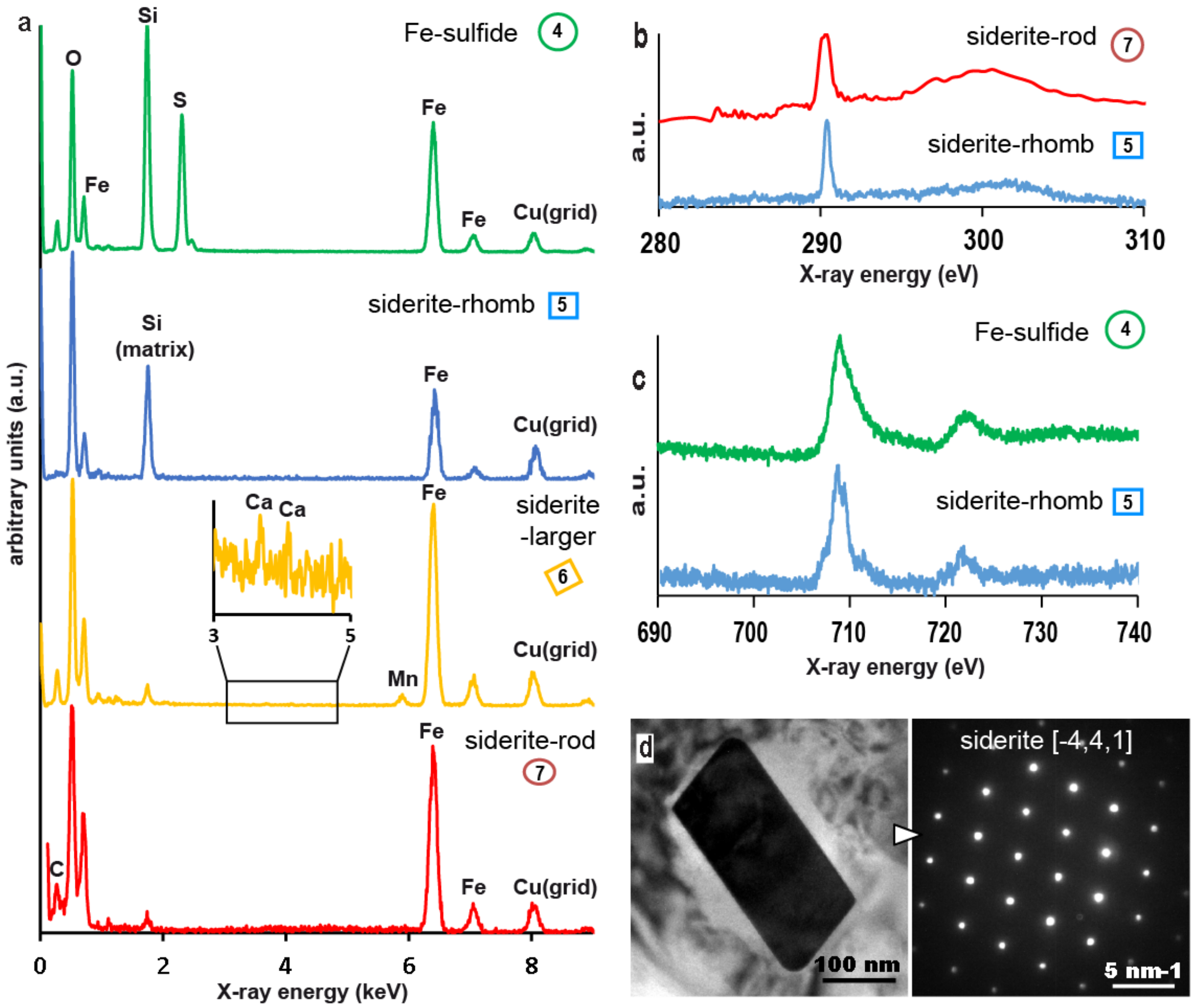


**Supplementary Figure 16 | Identification of siderite and $Fe^{2+}$-sulphides. a,** EDXS spectra of the Fe-sulphide crystal numbered 4 in Fig. 4h and of the siderite crystals numbered 5-7 in Fig. 4. **b,** Carbon 1s edge XANES (red) and EELS (blue) spectra identifying carbonate groups in siderites 5 and 7 of Figs. 4g and 4e. **c,** Fe $L_{2,3}$ edge EELS spectra of the siderite 5 in Fig. 4g and of the Fe-sulphide 4 in Fig. 4h showing a $Fe^{2+}$-only valence (band at 708.9 eV). **d**, TEM image and SAED along the [-4,4,1] zone axis of a rod-shaped siderite nanocrystal in boxed crystal of Supplementary Fig. 7c (*Gunflintia Grandis*).

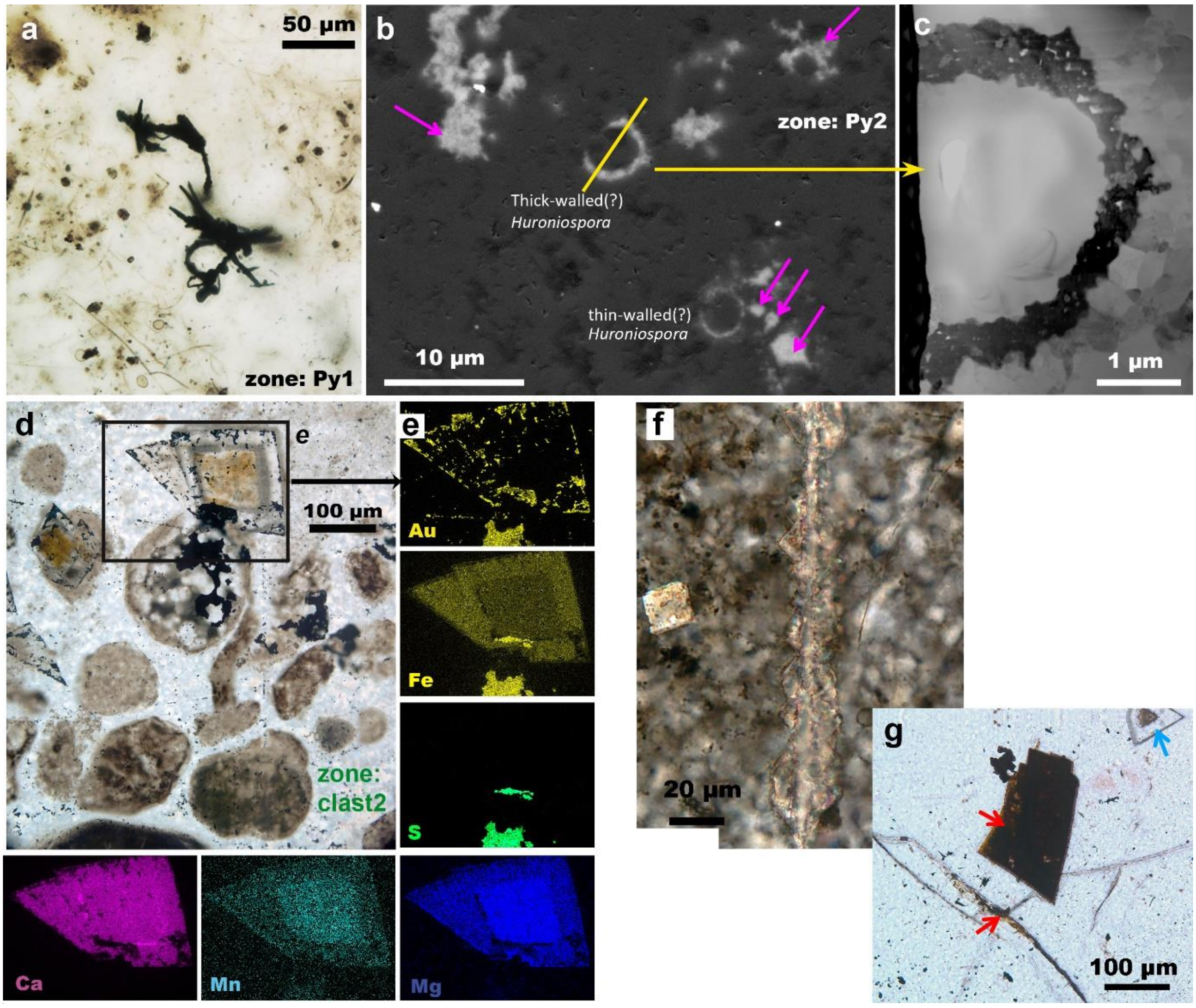


**Supplementary Figure 17 | Pyrite and ankerite in the stromatolite. a,** Region Py1 where *Gunflintia minuta* are replaced by pyrite (black). Yellow-to-brown organically-preserved microfossils are absent in the patch of chert surrounding these pyritised microfossils. **(b-c)** Pyritised *Huroniospora* of region Py2. **b**, SEM-BSE image showing pyrite (light grey) in quartz (dark). **c**, STEM bright-field image of the FIB section cut along the yellow line in (**b**) showing pyritised wall (dark) in quartz (light grey). **d,** Clastic grains of region Clast2, photomicrograph. Clastic grains are rounded to angular. Secondary ankerite rhombohedra cross-cut clasts (boxed zone). **e**, SEM EDXS maps of major elements in an ankerite rhombohedron, and pyrite inside the rounded clast (lower part of the boxed zone in **d**). Pyrite is also included in the ankerite rhombohedron. The iron-rich overgrowth on the carbonate rhombohedron shows no sign of Fe-oxidation. The gold (Au) patches are the remnants of a prior conductive coating (mostly removed by polishing) used for FIB experiments. **f,** vertical vein filled with ankerite (yellow-pink), and from which ankerite rhombohedra extend into the quartz (shades of grey) matrix at the margins of the vein. **g,** Open fracture with Fe-(oxy)hydroxide (red arrows) connected with an ankerite rhombohedron replaced by Fe-(oxyhydroxide). The ankerite rhombohedron that is not connected to the fracture (blue arrow) remained pristine. The (oxy)hydroxide nature was deduced by the absence of detectable Raman signal in conditions (1-5 mW laser power, 5 minutes acquisition time, 532 nm excitation ) that should readily detect hematite and magnetite.

## Supplementary Discussion

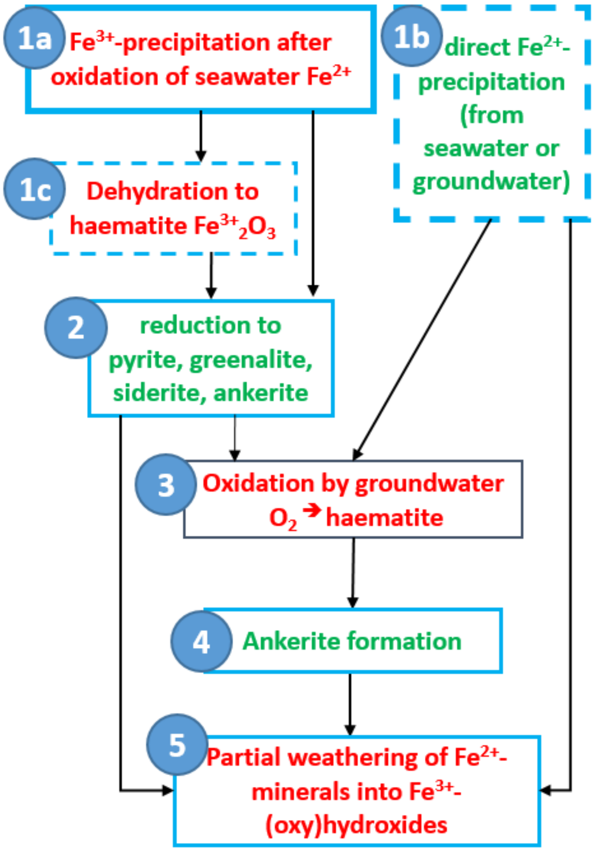


**Supplementary Figure 18 | <u>General paragenesis of Fe-minerals in the Gunflint Iron Formation.</u>** Circles indicate each stage. Red and green text indicate $Fe^{3+}$ and $Fe^{2+}$, respectively.

The global Fe-mineral paragenesis in the Gunflint Iron Formation can be summarized as follows. Stage (1a) of Supplementary Fig. 18 is indicated by Fe isotope compositions depleted in light Fe-isotopes throughout the Gunflint Iron Formation[5]. In Stage (1b) initial precipitation as $Fe^{2+}$-minerals could only have enriched the precipitates in light Fe-isotopes[6] – this was rarely observed[5], indicating that initial $Fe^{2+}$-precipitation was minor compared to $Fe^{3+}$-precipitation. $Fe^{2+}$-deposition followed by oxidation by groundwater $O_2$ (Ref.[7]) should preserve light Fe-isotope enrichments through quantitative transfer of iron from precursor mineral to product[8]. Light Fe-isotope enrichments are scarce in the Gunflint, indicating that a large fraction of the bulk-rock iron precipitated initially as $Fe^{3+}$-minerals from a large (buffered isotope composition) reservoir, i.e. ferruginous seawater rather than groundwater[5]. Indeed, precipitation of $Fe^{3+}$-minerals by groundwater should have rapidly enriched remaining groundwater $Fe^{2+}$ in the light isotopes[9], allowing for the subsequent formation of deposits with light Fe-isotope enrichments. In stage (1c) Some of this primary haematite may have been preserved[5], escaping reduction by e.g. organic matter in stage (2). Stage (2) could have proceeded through microbial and/or thermal oxidation of organic matter[10,11]. The depletion in light Fe-isotopes associated with $Fe^{3+}$-precipitation of stage (1a) could have been preserved through quantitative transfer of iron from precursor to products as observed in Gunflint rocks preserving stage (2) $Fe^{2+}$-minerals and consistent with the general absence of rocks enriched in light Fe-isotopes through Fe-reduction[5]. All $Fe^{3+}$-rich microfossil-rich rocks of the Gunflint should have formed some $Fe^{2+}$-minerals through thermal oxidation of the available organic matter as they suffered sufficient burial temperatures >150-170°C[10,12]. Stage (3) concerns microfossiliferous Gunflint samples where iron is only present as

haematite[5,7] and implies an additional stage (3) of oxidation of organic matter (all types of microfossils) and $Fe^{2+}$-minerals by groundwater[7]. This should have preserved the Fe-isotope signature[8] of stages (1) & (2). Locally, stage (2) assemblages (e.g. the studied sample) where preserved from (3) [5,7,13]. Stage (4) Ankerite results of late stage Fe-migration[14], as indicated by (Supplementary Fig. 17 and *Ref.*[13]): i) cross cutting of chert, ii) replacement of greenalite and siderite, iii) dominant occurrence in veins, and iv) systematic coarse crystal size and chemical zonation in veins and dispersed rhombohedra. Stage (5) affected only the Fe-minerals that are cut across by open cracks and must be recent because weathered cracks are open and Fe-(oxy)hydroxydes should have dehydrated (stage 1c) during burial.

Our stromatolite sample has been affected by stages in blue boxes and, possibly, by stages in discontinuous boxes of Supplementary Fig. 18. Stages (1b) and/or (1a±1c+2) formed the observed greenalite+siderite+pyrite. $Fe^{3+}$-oxidation stage (1a) is indicated by Fe-isotopes in haematitic stromatolites of the Gunflint as well as in deeper-water facies, suggesting that $Fe^{3+}$-oxidation could have proceeded in all facies of the Gunflint. Our sample was unaffected by stage (3) oxidation (indicated by preservation of organic matter, nanoscale $Fe^{2+}$-minerals, absence of haematite). At the sampled locality, lateral transitions between haematitic (stage 3) and preserved (organic-rich) stromatolites occur on the sub-meter scale[7,13]. This argues that the preserved stromatolite we studied (and other organic-rich stromatolites) could have accreted in similar Fe-oxidizing conditions than its haematitic counterparts at the Schrieber locality where Fe-isotope signatures of stage (1) are recorded. In our sample, stage (4) ankerite is absent in microfossils that preserve stage (2) Fe-minerals, indicating that it formed from distinct, Ca- and Mg- enriched fluids.

The following section presents five schematics (Supplementary Figs. 19-23) of the scenario discussed in the main text for the origin and fate of Fe-minerals in order to explain the presence and crystal chemistry of the assemblage of greenalite + siderite + Fe-sulphides in Gunflint Iron Formation microfossils.

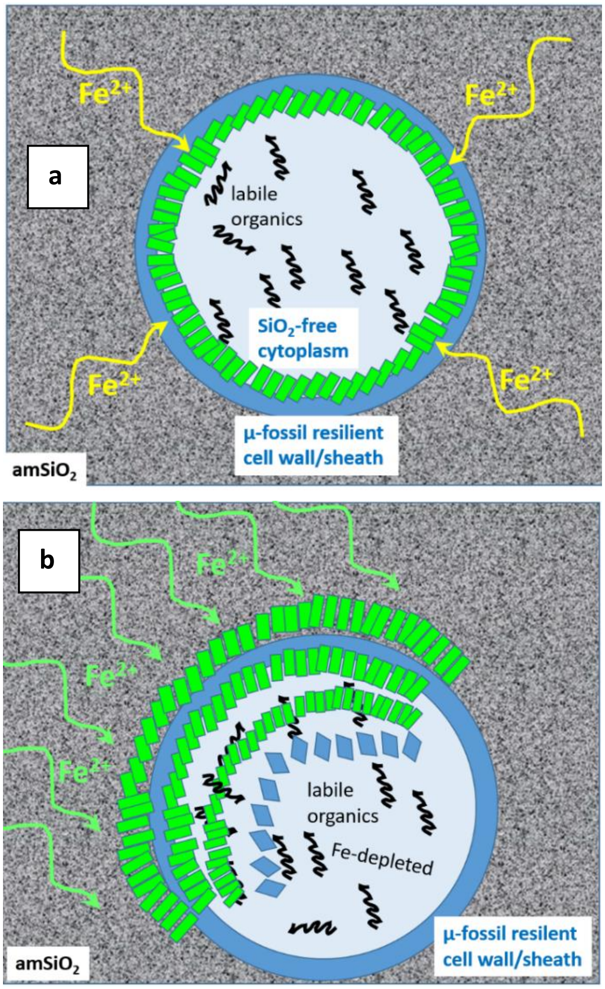


**Supplementary Figure 19 | Cells, encrusted only externally by amorphous $SiO_2$, have a decaying cytoplasm favoring post-mortem intra-microfossil mineralization through diffusion of externally-sourced Fe. a,** Illustration of the case where $Fe^{2+}$ diffusion is associated with convection, which allows aqueous $Fe^{2+}$ (or colloidal $Fe^{3+}$) to reach the decaying cell in an isotropic fashion. In this case, the wall of the dead cell would act as a double diffusion boundary where externally-sourced $Fe^{2+}$-fluids meet the organic-rich conditions of the cell wall and/or of the decaying intracellular content; both conditions could favor precipitation of iron. Precipitation should thus start at this diffusion boundary, and *post-mortem* $Fe^{2+}$ or $Fe^{3+}$ mineralization should encrust the inner and/or outer surface of cell walls first[15,16] and not only scatter crystals at the center of cells. This is supported by experimental evidence and observation of younger fossils, as listed in the main text. **b,** Illustration of the case of an anisotropic, convection-free diffusion of $Fe^{2+}$, which belongs to Liesegang-band formation processes. Similar to the diffusion+convection process, Fe-precipitation should occur at the diffusion boundary and/or at regular distances from this boundary. Fe-precipitation is expected to preferentially occur on the parts of the microfossils that are oriented toward the $Fe^{2+}$-diffusion front. Moreover, Fe-minerals of distinct compositions are expected to form in distinct bands (e.g. bands of greenalite separated from bands of siderite) due to contrasting saturation states created at the limits of the diffusion boundary[17]. Such banded Fe-precipitates have been observed in petrified wood, where bands cross-cut cell walls and are associated with dark Fe-mineral linings on the cell walls (Fig. 31 in *Ref.*[18]). However, no mineralogical control is observed on the spatial distribution of Fe-minerals in Gunflint microfossils: greenalite and siderite are homogeneously mixed at the center of microfossils. Moreover, no spatial correlation appears between Fe-minerals and the main diffusion boundary that is the cell wall. Therefore, these models of abiotic Fe-mineralization associated are difficult to reconcile with the microfossils mineralized by greenalite+siderite with only scattered intra-microfossil Fe-minerals and no minerals near/at the wall boundary.

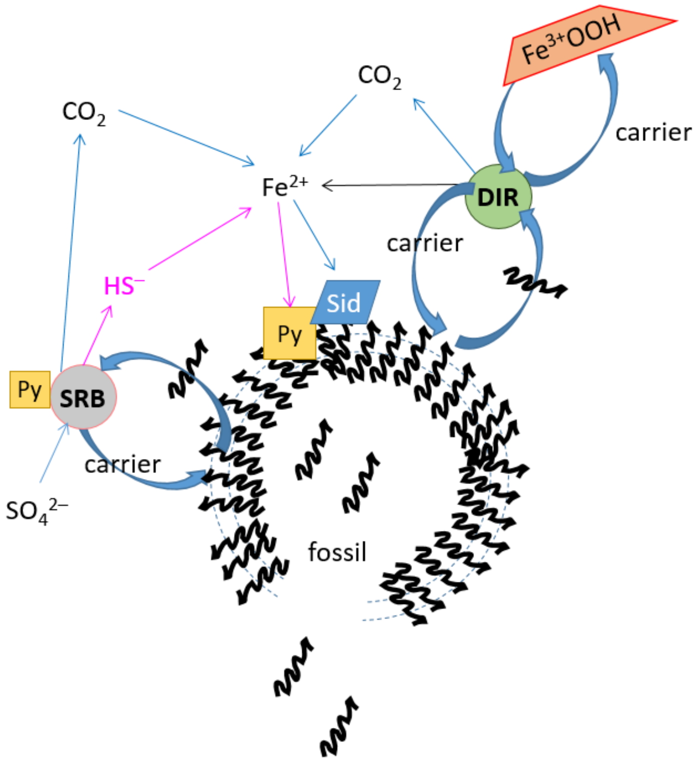


**Supplementary Figure 20 | Pyritization by Sulphate Reducing Bacteria (SRB) with/without Dissimilatory Iron Reducing (DIR) bacteria.** Model modified from the conclusions of *Ref.*[19] to add possible DIR as the source of $Fe^{2+}$ for pyritization. In these fossils, the organic matter is replaced by pyrite (Py), the product of heterotrophic SRB (possibly coupled with heterotrophic DIR) metabolism. SRB and DIR both oxidize organic matter (into $CO_2$) as a means to reduce sulfate (to $HS^-$) and $Fe^{3+}$-minerals (to $Fe^{2+}$), respectively. SRB and DIR have exoenzymes (carriers) that allow extraction and transport of organic "fuel", and DIR have carrier molecules (siderophores) for extraction and transport of $Fe^{3+}$ (*Ref.*[20]). The products of these metabolisms ($CO_2$, $HS^-$ and $Fe^{2+}$) can form pyrite and/or siderite, as well as greenalite in presence of silicic acid (*Ref.*[11]). In this model, almost all the organic molecules (black chains) of the fossils, including the resilient sheath/wall polysaccharides, are available for heterotrophic consumption, as indicated by the low abundance of organic matter in these fossils [*Ref.*[19] and Supplementary Fig. 17a-c]. The localization of pyrite replacing the organic walls of the fossil can be explained e.g. by $Fe^{2+}$ adsorption onto remnants of polysaccharide wall[21,22] during pyritization. Pyrite is abundant outside the microfossils, as scattered crystals and overgrowths of pyritized microfossils (pink arrows in Supplementary Fig. 17b) and encrustation of the heterotrophic SRB (*Ref.*[19]). Supplementary Figs. 17b-c show the absence of intracellular pyrite and the general absence of Fe-minerals inside the pyritized cells, suggesting that intracellular contents did not favor pyritization. Altogether, the mineralization pattern and the replacement of organic matter in pyritized microfossils strongly contrast with organically-preserved microfossils with intracellular greenalite+siderite, indicating that the latter could not form from oxidation (stage 3 or 5) of the former.

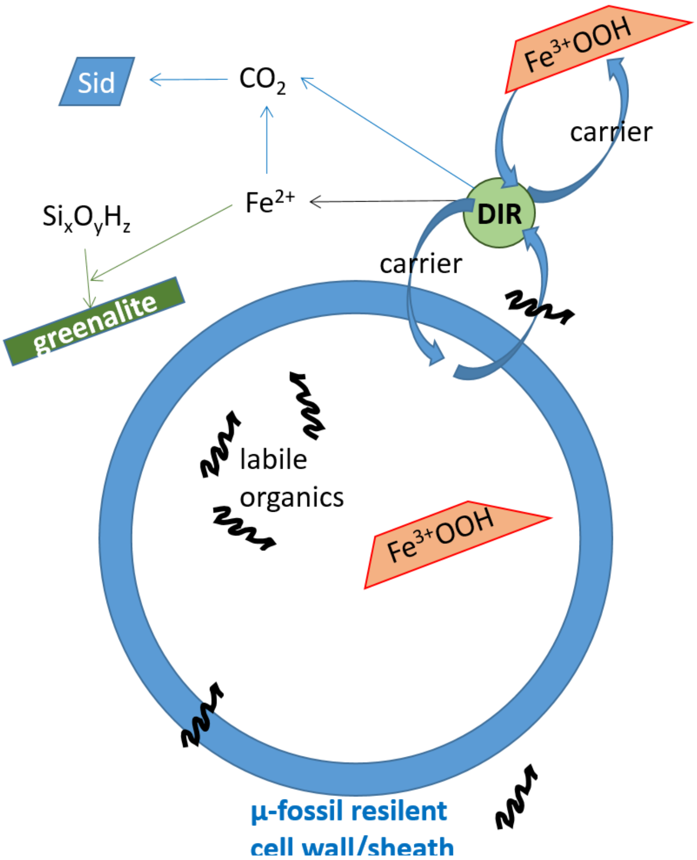


**Supplementary Figure 21 | Bacterial Dissimilatory Iron Reduction (DIR) of intra and/or extracellular $Fe^{3+}$-minerals - case of a fossil cell wall that is not breached by DIR-bacteria.** Heterotrophic DIR bacteria can form greenalite, siderite and Fe-sulphides (*Ref.*[11]) from remote $Fe^{3+}$-minerals and organic molecules (*Ref.*[20]) extracted for example from decaying cells. First, the possibility that the microfossils themselves could represent DIR bacteria (which can precipitate intracellularly: *Ref.*[23]) is ruled out by their morphology (large, thick-walled spheres and filaments unknown in DIR bacteria). Second, organic matter is preserved in fossil sheaths, cell walls and inside microfossils. DIR-bacteria can produce catalytic exoenzymes (carrier) to reach intracellular content of the decaying cells and carry fuel for $Fe^{3+}$-respiration (*Ref.*[20]). Third, $Fe^{3+}$, being highly insoluble, must be sourced in minerals outside or inside microfossils; siderophores (carriers, *Ref.*[20]) can also reach and carry $Fe^{3+}$ from inside and outside the dead cells. In this model, both $Fe^{2+}$ and $CO_2$, that can drive the precipitation of greenalite and/or siderite (Sid), are produced by the DIR bacteria. In this model, the DIR bacteria do not breach the cell wall and penetrate physically the microfossils, which is consistent with the wall preservation observed in the microfossils. Therefore, irrespective of the localization of the source of $Fe^{3+}$, $Fe^{2+}$ precipitation should be favored outside the microfossils where DIR bacteria produce $Fe^{2+}$ and $CO_2$. However, outside microfossils, possible greenalite is extremely small and scarce and siderite has not been observed. Assuming that the $Fe^{2+}$ produced outside microfossils by DIR diffuses and finds better conditions to precipitate inside than outside microfossils recalls the model of Supplementary Fig. 19, which has been discussed above.

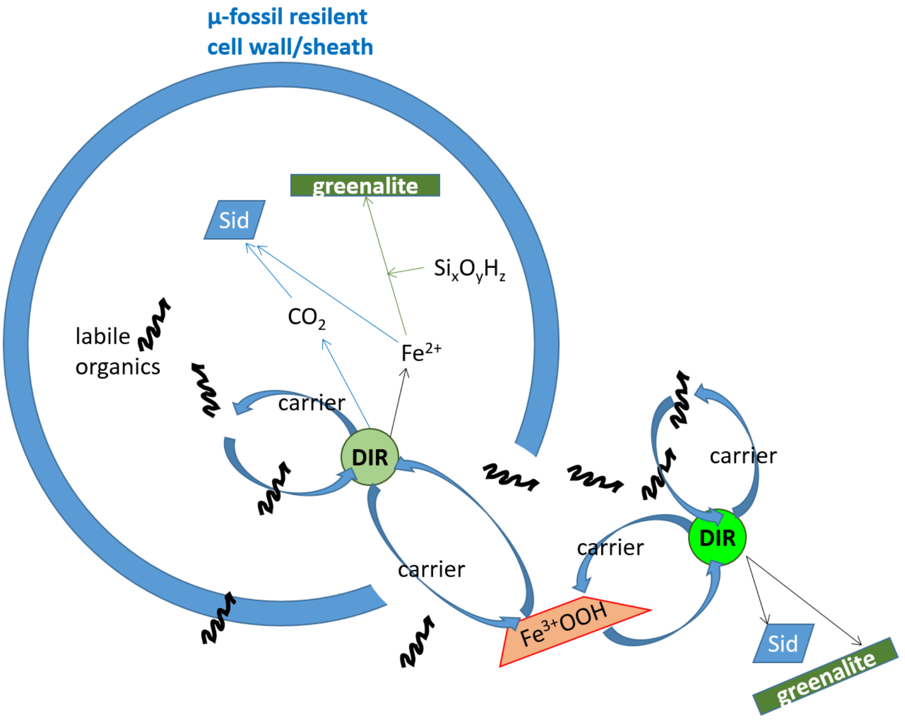


**Supplementary Figure 22 | Heterotrophic dissimilatory iron reduction (DIR) of intra and/or extracellular $Fe^{3+}$-minerals - case of a fossil cell wall that is physically breached by DIR-bacteria.** Here, DIR-bacteria that breach the wall respire labile (e.g. amino acids, proteins, nucleic acids,…) organics inside the dead cell where it can form *post-mortem* intra-microfossil Fe-minerals (e.g. greenalite and siderite) even if $Fe^{3+}$ is sourced outside microfossils. However, the physical breach that allowed penetration of DIR bacteria inside the microfossil cell must leak the intracellular organic compounds that fuel DIR. In turn, extracellular DIR bacteria thriving outside the decaying cells are fed by leaked compounds outside the cells, and if the $Fe^{3+}$-mineral source is only outside microfossil, then $Fe^{2+}$-mineralization is expected to occur as much outside as inside microfossils. The assemblage of greenalite and siderite would dominantly have formed outside and/or on the surface (Supplementary Fig. 19) of microfossils, which is not the case in our sample, hence refuting this model as the origin of the large concentrations of iron in thick-walled *Hurosniospora* and Type 2 *Gunflintia*. In addition, physical breaching of the cell wall by heterotrophs prior to encapsulation by silica is difficult to reconcile with the 3D preservation of the microfossils. Indeed, in modern siliceous environments, 3D preservation is allowed by a first step of external (and/or external + internal) silicification of unbreached cells followed by cytoplasm degradation and secondary silica infilling (*Ref.*[24]). Encapsulation by silica would drastically reduce physical access to the decaying cells by DIR and other heterotrophic bacteria, consistent with the molecular preservation of Gunflint microfossils (*Ref.*[12]).

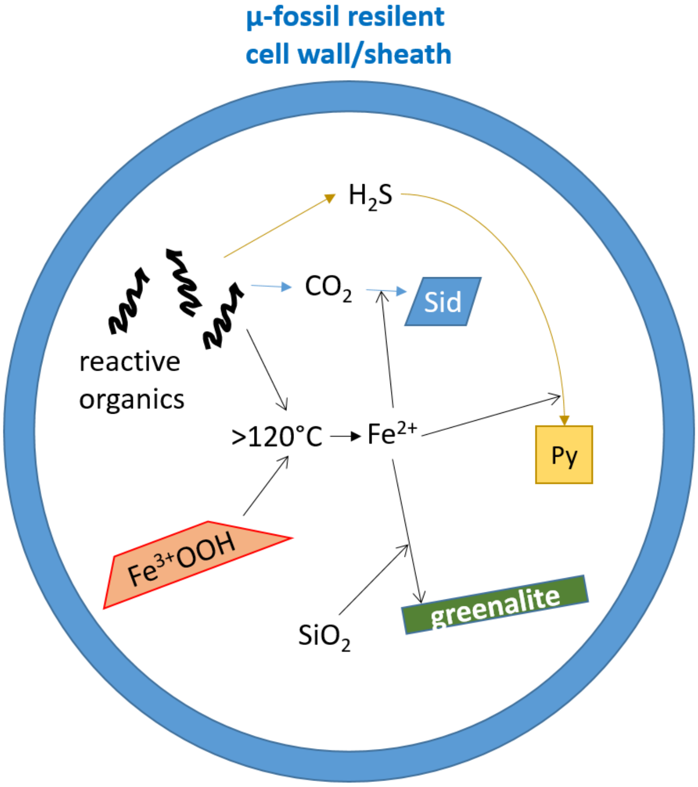


**Supplementary Figure 23 | *In situ*, thermal reduction of intra-microfossil $Fe^{3+}$-minerals.** In this model, oxidation of organic matter (ultimately, catagenesis to $CO_2$) is coupled with the reduction of $Fe^{3+}$-bearing minerals (here simplified as FeOOH) and catalyzed by thermal energy[10]. Siderite can form from the produced $Fe^{2+}$ and the produced $CO_2$[10]. Greenalite can similarly form from the reaction of the produced $Fe^{2+}$ with the silica matrix[25] and $H_2O$ from fluids and/or organic matter catagenesis. The extremely scarce nanoscale $Fe^{2+}$-sulphides can form by catagenetic desulphurization of organic matter producing $H_2S$ to react with $Fe^{2+}$ (*Ref.*[26]), consistent with systematic embedding of nanoscale sulphides in organic matter (Fig. 4). Because reactive organic molecules are mobile during this stage, whereas precursor $Fe^{3+}$-minerals are not until they are reduced (by their reaction with organic matter), localized $Fe^{2+}$-minerals can form by *in situ* replacement of $Fe^{3+}$-minerals. At this stage, silicification already occurred, amorphous $SiO_2$ was largely recrystallized to quartz, which limited the diffusion of OM and metamorphic products, favoring *in situ* replacement and explaining the scarcity of greenalite and absence of siderite outside microfossils. This contrasts with pyritization (Supplementary Fig. 20) and bacterial (heterotrophic) iron reduction (Supplementary Figs. 21-22) that require chemical exchange between microfossils and heterotrophic bacteria through a more permeable matrix. Some of the internal organic matter now observed inside the microfossils (Figs. 1-2, Supplementary Fig. 6) could correspond to relics of the cytoplasmic contents that could have fueled iron reduction. Finally, these thermal diagenetic reactions require a localized, mostly intracellular source of iron to explain the localization of greenalite+siderite+sulphides. Some siderites display rod shapes (Figs. 4a-f, Supplementary Fig. 16d) instead of their normal rhombohedral shapes (Fig. 4g), consistent with *in situ* recrystallization of rod-shaped $Fe^{3+}$-(oxyhydr)oxides. Moreover, similar to Supplementary Fig. 19, externally sourced $Fe^{2+}$ (e.g. from pore fluids) or colloids of $Fe^{3+}$ and organic molecules would lead to preferential precipitation of Fe-minerals in the matrix surrounding microfossils and/or at the cell wall interface, but not only at the center of microfossils.

## Supplementary References


1 Boal, D. & Ng, R. Shape analysis of filamentous Precambrian microfossils and modern cyanobacteria. *Paleobiology* **36**, 555–572 (2010).

2 Guggenheim, S. & Eggleton, R. A. Modulated crystal structures of greenalite and caryopilite: a system with long range, in plane structural disorder in the tetrahedral sheet. *Can. Mineral.* **36**, 163-179 (1998).

3 Bourdelle, F. *et al.* Quantification of the ferric/ferrous iron ratio in silicates by scanning transmission X-ray microscopy at the Fe L2,3 edges. *Contrib. Mineral. Petr.* **166**, 423-434 (2013).

4 Garvie, L. A. J., Zega, T. J., Rez, P. & Buseck, P. R. Nanometer-scale measurements of Fe3+/ΣFe by electron energy-loss spectroscopy: a cautionary note. *Am. Miner.* **89**, 1610–1616 (2004).

5 Planavsky, N. *et al.* Iron-oxidizing microbial ecosystems thrived in late Paleoproterozoic redox-stratified oceans. *Earth Planet. Sc. Lett.* **286**, 230-242 (2009).

6 Dauphas, N. & Rouxel, O. Mass spectrometry and natural variations of iron isotopes. *Mass Spectrom. Rev.* **25**, 515–550 (2006).

7 Shapiro, R. S. & Konhauser, K. O. Hematite-coated microfossils: primary ecological fingerprint or taphonomic oddity of the Paleoproterozoic? *Geobiology* **13**, 209-224 (2015).

8 Markl, G., von Blanckenburg, F. & Wagner, T. Iron isotope fractionation during hydrothermal ore deposition and alteration. *Geochim. Cosmochim. Ac.* **70**, 3011–3030 (2006).

9 Teutsch, N., von gunten, U., Porcelli, D., Cirpka, O. A. & Halliday, A. N. Adsorption as a cause for iron isotope fractionation in reduced groundwater. *Geochim. Cosmochim. Ac.* **69** 4175–4185 (2005).

10 Koëhler, I., Konhauser, K.O., Papineau, D., Bekker, A., Kappler, A. Biological carbon precursor to diagenetic siderite with spherical structures in iron formations. *Nat. Commun.* **4**, doi:10.1038/ncomms2770 (2013).

11 Percak-Dennett, E. M. *et al.* Iron isotope fractionation during microbial dissimilatory iron oxide reduction in simulated Archaean seawater. *Geobiology* **9**, 205-220 (2011 ).

12 Alleon, J. *et al.* Molecular preservation of 1.88 Ga Gunflint organic microfossils as a function of temperature and mineralogy. *Nat. Commun.* **7:11977**, doi: 10.1038/ncomms11977 (2016).

13 Barghoorn, E. S. & Tyler, S. A. Microorganisms from the Gunflint Chert. *Science* **147**, 563-575 (1965).

14 Floran, R. J. & Papike, J. J. Petrology of the low-grade rocks of the Gunflint Iron-Formation, Ontario-Minnesota. *Geol. Soc. Am. Bull.* **86**, 1169-1190 (1975).

15 Parenteau, M. N. & Cady, S. L. Microbial biosignatures in iron-mineralized phototrophic mats at Chocolate Pots hot springs, Yellowstone national park, United States. *Palaios* **25**, 97-111 (2010).

16 Wacey, D. *et al.* Enhanced cellular preservation by clay minerals in 1 billion-year-old lakes. *Sci. Rep.* **4**, doi:10.1038/srep05841 (2014).

17 Cartwright, J. H. E., García-Ruiz, J. M. & Villacampa, A. I. Pattern formation in crystal growth: Liesegang rings. *Comput. Phys. Commun.* **121–122**, 411–413 (1999).

18 Buurman, P. Mineralization of fossil wood. *Scripta Geol.* **12**, 1-43 (1972).

19 Wacey, D. *et al.* Nanoscale analysis of pyritized microfossils reveals differential heterotrophic consumption in the ~1.9-Ga Gunflint chert. *Proc. Natl. Acad. Sci. U.S.A.* **110**, 8020-8024 (2013).

20 Melton, E. D., Swanner, E. D., Behrens, S., Schmidt, C. & Kappler, A. The interplay of microbially mediated and abiotic reactions in the biogeochemical Fe cycle. *Nat. Rev. Microbiol.* **12**, 797-808 (2015).

21 Degens, E. T., Watson, S. W. & Remsen, C. C. Fossil membranes and cell wall fragments from a 7000-year-old Black Sea sediment. *Science* **168**, 1207-1208 (1970).

22 Ferris, F. G., Fyfe, W. S. & Beveridge, T. J. Metallic ion binding by Bacillus subtilis: Implications for the fossilization of microorganisms. *Geology* **16**, 149-152 (1988).

23 Glasauer, S., Langley, S. & Beveridge, T. J. Intracellular Iron minerals in a dissimilatory Iron-reducing bacterium. *Science* **295**, 117-119 (2002).

24 Campbell, K. A. *et al.* Tracing biosignature preservation of geothermally silicified microbial textures into the geological record. *Astrobiology* **15**, 858-882 (2015).

25 Planavsky, N. *et al.* Iron isotope composition of some Archean and Proterozoic iron formations. *Geochim. Cosmochim. Ac.* **80**, 158-169 (2012).

26 Aplin, A. C. & Macquaker, J. H. S. C-S-Fe Geochemistry of Some Modern and Ancient Anoxic Marine Muds and Mudstones. *Philos. Transact. A Math. Phys. Eng. Sci.* **344**, 89-100 (1993)
